# Dual-sgRNA CRISPR/Cas9 knockout of PD-L1 in human U87 glioblastoma tumor cells inhibits proliferation, invasion, and tumor-associated macrophage polarization

**DOI:** 10.1038/s41598-022-06430-1

**Published:** 2022-02-14

**Authors:** Javier Fierro, Jake DiPasquale, Joshua Perez, Brandon Chin, Yathip Chokpapone, An M. Tran, Arabella Holden, Chris Factoriza, Nikhi Sivagnanakumar, Rocio Aguilar, Sarah Mazal, Melissa Lopez, Huanyu Dou

**Affiliations:** 1 Department of Molecular and Translational Medicine, Paul L. Foster School of Medicine, Texas Tech University Health Science Center, 5001 El Paso Drive, El Paso, TX 79905-2827 USA; 2 Graduate School of Biomedical Sciences, Texas Tech University Health Science Center, El Paso, TX USA

**Keywords:** CNS cancer, Gene regulation

## Abstract

Programmed death ligand 1 (PD-L1) plays a key role in glioblastoma multiforme (GBM) immunosuppression, vitality, proliferation, and migration, and is therefore a promising target for treating GBM. CRISPR/Cas9-mediated genomic editing can delete both cell surface and intracellular PD-L1. This systemic deliverable genomic PD-L1 deletion system can be used as an effective anti-GBM therapy by inhibiting tumor growth and migration, and overcoming immunosuppression. To target PD-L1 for CRISPR/Cas9 gene editing, we first identified two single guide RNA (sgRNA) sequences located on PD-L1 exon 3. The first sgRNA recognizes the forward strand of human PD-L1 near the beginning of exon 3 that allows editing by Cas9 at approximately base pair 82 (g82). The second sgRNA recognizes the forward strand of exon 3 that directs cutting at base pair 165 (g165). A homology-directed repair template (HDR) combined with the dual-sgRNAs was used to improve PD-L1 knockout specificity and efficiency. sgRNAs g82 and g165 were cloned into the multiplex CRISPR/Cas9 assembly system and co-transfected with the HDR template in human U87 GBM cells (g82/165 + HDR). T7E1 analysis suggests that the dual-sgRNA CRISPR/Cas9 strategy with a repair template was capable of editing the genomic level of *PD-L1*. This was further confirmed by examining PD-L1 protein levels by western blot and immunofluorescence assays. Western blot analysis showed that the dual-sgRNAs with the repair template caused a 64% reduction of PD-L1 protein levels in U87 cells, while immunostaining showed a significant reduction of intracellular PD-L1. PD-L1 deletion inhibited proliferation, growth, invasion and migration of U87 cells, indicating intracellular PD-L1 is necessary for tumor progression. Importantly, U87 cells treated with g82/165 + HDR polarized tumor-associated macrophages (TAM) toward an M1 phenotype, as indicated by an increase in TNF-α and a decrease in IL-4 secretions. This was further confirmed with flow cytometry that showed an increase in the M1 markers Ly6C + and CD80 +, and a decrease in the M2 marker CD206 + both in vitro and in vivo. Utilizing dual-sgRNAs and an HDR template with the CRISPR/Cas9 gene-editing system is a promising avenue for the treatment of GBM.

## Introduction

Glioblastoma multiforme (GBM) is a very aggressive and deadly brain tumor. The survival rate has not changed over the past decade, with most patients dying within 1–2 years after diagnosis^1–5^. Patients with GBM undergo a series of treatments including surgery, radiation, and chemotherapy, but these treatments do not significantly improve survival rate or quality of life^6,7^. Recently, immunotherapies have demonstrated promising results for tumor eradication by upregulating innate and adaptive immune responses^8–10^. However, GBM patient outcome using a combination of immunotherapies are still inadequate^11–13^.

Programmed death ligand 1 (PD-L1) is a key player involved in both tumor progression and tumor-associated immune suppression. PD-L1 expression on the surface of immune cells and tumor cells generates immune resistance upon binding to programmed cell death protein 1 (PD-1). On the other hand, intracellular PD-L1 promotes cell growth, proliferation, and migration. Highly aggressive GBM tumors express higher levels of PD-L1, which is correlated with worse survival outcomes^14–17^. Thus, PD-L1 is a therapeutic target for many cancers including GBM^18–21^. Given that inhibitors and monoclonal antibodies only block cell surface PD-L1, uninhibited intracellular PD-L1 can still diminish immunotherapeutic efficacy. This occurs by PD-L1 translocating to the cell surface to evade immune attack, and by promoting tumor progression. Understanding how PD-L1 mediates immunosuppression through cell surface expression, and how PD-L1 contributes to GBM progression through cytoplasm and nuclear expression, can aid in the development of an effective treatment for GBM. Knock out of both cell surface and intracellular PD-L1 provides a novel strategy that has the potential for anti-GBM therapy.

Genetic deletion of tumor associated genes using clustered regularly interspaced short palindromic repeats (CRISPR) with CRISPR associated protein-9 nuclease (CRISPR/Cas9) has great potential in anti-cancer therapy^22–25^. CRISPR/Cas9 gene-editing relies on the guidance of a single-guide RNA (sgRNA)^26,27^ to disrupt targeted genes^28–31^. However, target specificity (off-target effects) and gene-editing efficiency are still major challenges for applying CRISPR/Cas9 technology in biomedical and clinical applications^32–35^. Off-target effects result from a sgRNA recognizing an unintended DNA sequence that leads to cutting outside of the target gene^36–39^. Further, the Cas9 nuclease enzyme creates a double strand break (DSB) within the target gene that the cell can repair by non-homologous end joining (NHEJ) or homology directed repair (HDR) pathways^40–43^. Importantly, repair of genomic DNA by these pathways can lead to the production of a null mutation. Finally, each cell will repair the DSB created by Cas9 differently, leading to a mosaic of mutations within a single cell population.

In this study, we identified two sgRNAs and cloned them into the multiplex CRISPR/Cas9 assembly system^44^. This plasmid was co-transfected with a synthetic single stranded HDR repair template for knockout of PD-L1. Human GBM U87 cells were treated with the dual-sgRNA CRISPR/Cas9 + HDR gene-editing system to evaluate cutting specificity and PD-L1 knockout efficiency. We found that this dual-sgRNA CRISPR/Cas9 gene-editing strategy with the repair template was more efficient at knocking out PD-L1 in U87 cells than either sgRNA alone or dual-sgRNAs without the repair template. Interestingly, significant intracellular PD-L1 deletion prevented GBM U87 cell migration and proliferation compared to control cells. Our results suggest that besides PD-L1’s immunosuppressive function, PD-L1 is critical for tumor progression and migration. Interestingly, the loss of PD-L1 in U87 GBM cells polarizes tumor-associated macrophages (TAMs) toward an M1 phenotype compared to control cells. This suggests the loss of PD-L1 on U87 cells leads to the activation of macrophages in an in vitro GBM model. Employing the dual-sgRNA + HDR strategy in vivo in the absence of a GBM tumor was also able to knockdown PD-L1 in the spleen, which led to the polarization of M1 macrophages. This confirms previous results that the loss of PD-L1 is important for regulating immune functions. This is the first time CRISPR/Cas9 technology has been used with dual-sgRNAs and a repair template to target PD-L1 in human glioblastoma cells. Dual-sgRNA CRISPR/Cas9 + HDR to knockout PD-L1 in U87 cells shows great potential for the treatment of GBM.

## Results

### Identification of dual-sgRNAs and design of the synthetic HDR template

**Figure 1:**
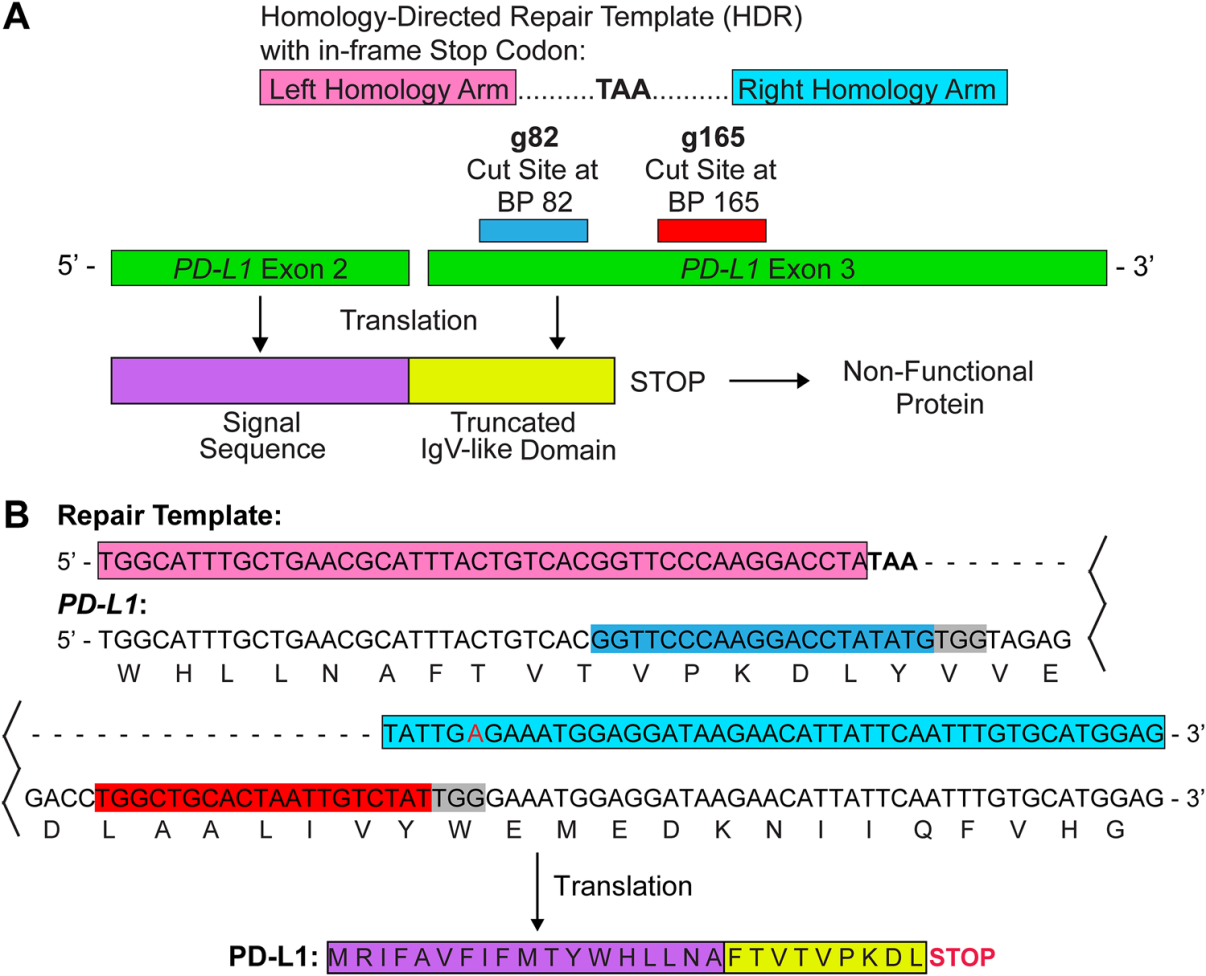
Schematic of dual-sgRNAs and a HDR template for the CRISPR/Cas9 system. (**A**) sgRNA 82 (g82; cyan) and sgRNA 165 (g165; red) were chosen for *PD-L1* gene-editing due to their high on-target and low off-target properties. g82 recognizes the forward strand of *PD-L1* exon 3 (green) and cuts at approximately base pair (bp) 82. g165 recognizes the forward strand of exon 3 and cuts at bp 165. To promote *PD-L1* gene-editing, a homology directed repair template (HDR) was designed to incorporate an in-frame stop codon (TAA). The left (pink) and right (light blue) homology arms recognize 45 bps of sequence on either side of the g82 and g165 cut sites, respectively. After gene-editing, only the signal sequence (purple) and a truncated IgV-like domain (yellow) of PD-L1 are translated. This leads to the production of a non-functional PD-L1 protein. (**B**) DNA sequence of *PD-L1*, the repair template, sgRNA’s, and the translated protein sequence after gene editing. Colored sequences in B match sequences labeled in (**A**). Additionally, the red (**A**) in the right homology arm indicates a single bp modification in the PAM sequence (grey) to prevent cutting of the repair template by the Cas9 enzyme.

We first identified two sgRNA sequences that specifically knockout *PD-L1* with high on-target and low off-target properties, with the most likely off-target sequences found in non-coding regions (Supplemental Tables 1 and 2). The first sgRNA (g82) recognizes the forward strand of human *PD-L1* near the beginning of exon 3 and cuts at approximately base pair 82 (Fig. 1A). The second sgRNA (g165) recognizes the forward strand of exon 3 and cuts at base pair 165. sgRNAs g82 and g165 create an 83 bp deletion in the genomic sequence that leads to the production of a non-functional PD-L1 protein. However, cells can repair the deletion through NHEJ or HDR pathways that leads to inefficient PD-L1 deletion. To enhance gene-editing efficiency, we designed a HDR template that can precisely repair the double strand break. The HDR template contains 45 bps of homologous sequence upstream of cut site g82 and downstream of cut site g165 (Fig. 1A) that incorporates an in-frame stop codon (TAA). This aids in generating a non-functional PD-L1 protein containing only the signal sequence and a truncated IgV-like domain. It is important to note that we altered the PAM sequence in the right homology arm to prevent Cas9 from destroying the repair template (Fig. 1B).

Two sgRNA sequences were also identified for mouse PD-L1 that have high on-target and low off target properties (Supplemental Tables 3 and 4). The first sgRNA (g70) recognizes the forward strand of mouse *PD-L1* near the beginning of exon 3 and cuts at approximately base pair 70. The second sgRNA (g166) recognizes the reverse strand of exon 3 and cuts at base pair 166. sgRNAs g70 and g166 create a 96 bp deletion in the genomic sequence that leads to the production of a non-functional PD-L1 protein. A HDR template was also synthesized that contains 45 bps of homologous sequence upstream of cut site g70 and downstream of cut site g166 that incorporates an in-frame stop codon (TAA).

### Construction of the dual-sgRNA containing CRISPR/Cas9 plasmid

Next, each sgRNA was cloned into the multiplex CRISPR/Cas9 assembly system (Supplemental Fig. 1)^44^. We first cloned g82 and g165 into two separate CRISPR/Cas9 plasmids (Cas9-g82 and Cas9-g165; Supplemental Fig. 1A). This allowed both sgRNAs to be cloned into a single CRISPR/Cas9 plasmid by Golden Gate assembly (Cas9-g82/165; Supplemental Fig. 1B). GFP tagged Cas9-g82 and Cas9-g165 were also prepared for determining transfection efficiency^45^. All plasmids were sequenced to identify successfully assembled clones. Cas9-g82/165 co-transfected with the HDR template (Cas9-g82/165 + HDR) was employed for maximizing gene-editing of *PD-L1* while reducing off-target effects. Mouse guides g70 and g166 were also cloned into the multiplex CRISPR/Cas9 assembly system for use in in vivo studies.

### Gene-editing efficiency of Cas9-g82/165 + HDR in U87 cells

**Figure 2:**
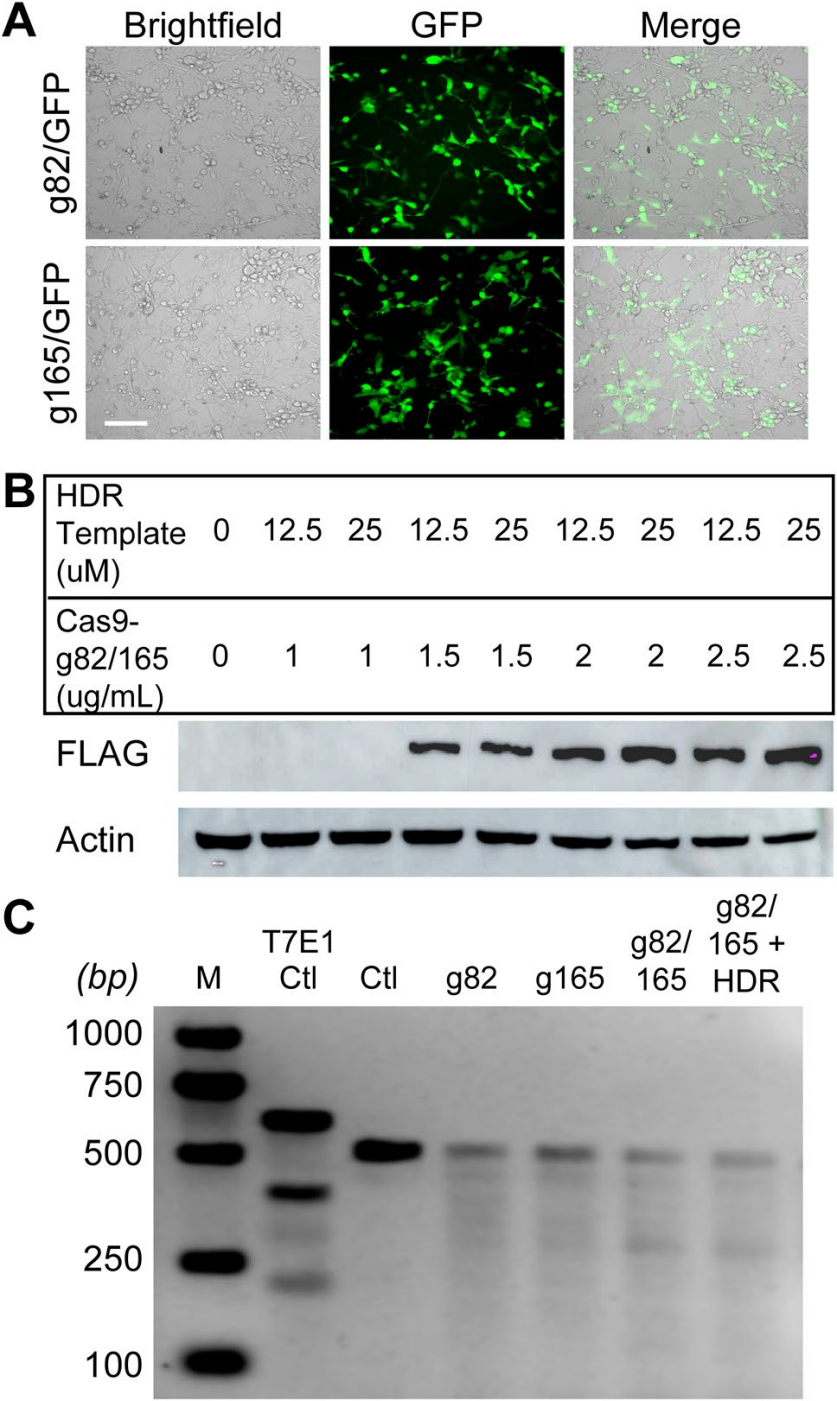
Transfection of CRISPR/Cas9 plasmids and genomic editing efficiency in human U87 cells. (**A**) Transfection of GFP tagged Cas9-g82 and Cas9-g165 in U87 cells was evaluated by fluorescent microscopy. GFP expression indicates successful transfection of Cas9-g82/GFP and Cas9-g165/GFP in U87 cells at day 2. (**B**) Optimization of Cas9-g82/165 transfection. Cas9-g82/165 was transfected at different concentrations with either 12.5 µM or 25 µM of the HDR template, and Cas9 expression was evaluated by western blot analysis. Cas9 is fused with an N-terminal FLAG tag, and actin was used to evaluate protein levels. While concentrations above 1.5 ug/mL lead to Cas9 transfection, low toxicity was observed with a concentration of 1.5 ug/mL. (**C**) The T7E1 assay was performed to determine the efficiency of *PD-L1* gene-editing at the genomic level. Multiple bands for a single sample indicate successful gene editing by Cas9. A ladder (M; lane 1), and a positive control (T7E1 ctl; lane 2) were used to confirm the size of the uncut product (Ctl) and the efficacy of the assay, respectively. Gel electrophoresis indicates all CRISPR/Cas9 plasmids were able to edit *PD-L1* at the genomic DNA level, with an increased editing efficiency observed with Cas9-g82/165 + HDR. Scale bar = 50 um.

Transfection efficiency was first determined by examining GFP expression after treating U87 cells with Cas9-g82/GFP and Cas9-g165/GFP. Fluorescent microscopy revealed high GFP expression 2 days post transfection (Fig. 2A). To optimize transfection, U87 cells were treated with varying concentrations of both Cas9-g82/165 and the HDR template, and Cas9 expression was evaluated by western blot analysis (Fig. 2B and Supplemental Fig. 2). The FLAG tag is fused to the N terminus of Cas9, allowing visualization of Cas9 transfection efficiency. Successful transfection of Cas9-g82/165 was seen at 1.5ug/mL. Higher concentrations of Cas9-g82/165 caused greater toxicity as determined by visually inspecting cells and performing an MTT assay to determine cellular metabolic activity (data not shown). Toxicity was also observed when cells were transfected with the HDR template at a final concentration of 25 µM (data not shown). This data suggests that transfection of Cas9-g82/165 at a concentration of 1.5ug/mL co-transfected with the HDR template at a final concentration of 12.5 µM produced the most efficient transfection with low cytotoxic effects.

To evaluate the efficiency of PD-L1 gene-editing, we used the T7E1 assay (Fig. 2C). This assay uses primers to amplify a small region surrounding the target loci on the genomic DNA that is then denatured, re-annealed, and cut with T7 endonuclease 1. This enzyme only cuts mismatched heteroduplexed DNA, which indicates successful gene-editing. U87 cells were transfected with Cas9-g82, Cas9-g165, Cas9-g82/165 and Cas9-g82/165 + HDR. U87 cells transfected with lipofectamine only served as the control. Agarose gel electrophoresis revealed all CRISPR/Cas9 constructs were able to edit *PD-L1* at the genomic level, with the strongest editing efficiency seen in cells treated with Cas9-g82/165 + HDR (Fig. 2C). It is important to note that a larger band was seen in the control sample. As PD-L1 knockout has been shown to decrease cell growth and proliferation, it is to be expected that there would be less genomic DNA available as a result of the reduction in cell numbers after PD-L1 knockout compared to the control.

### Enhanced PD-L1 knockout by Cas9-g82/165 + HDR

**Figure 3:**
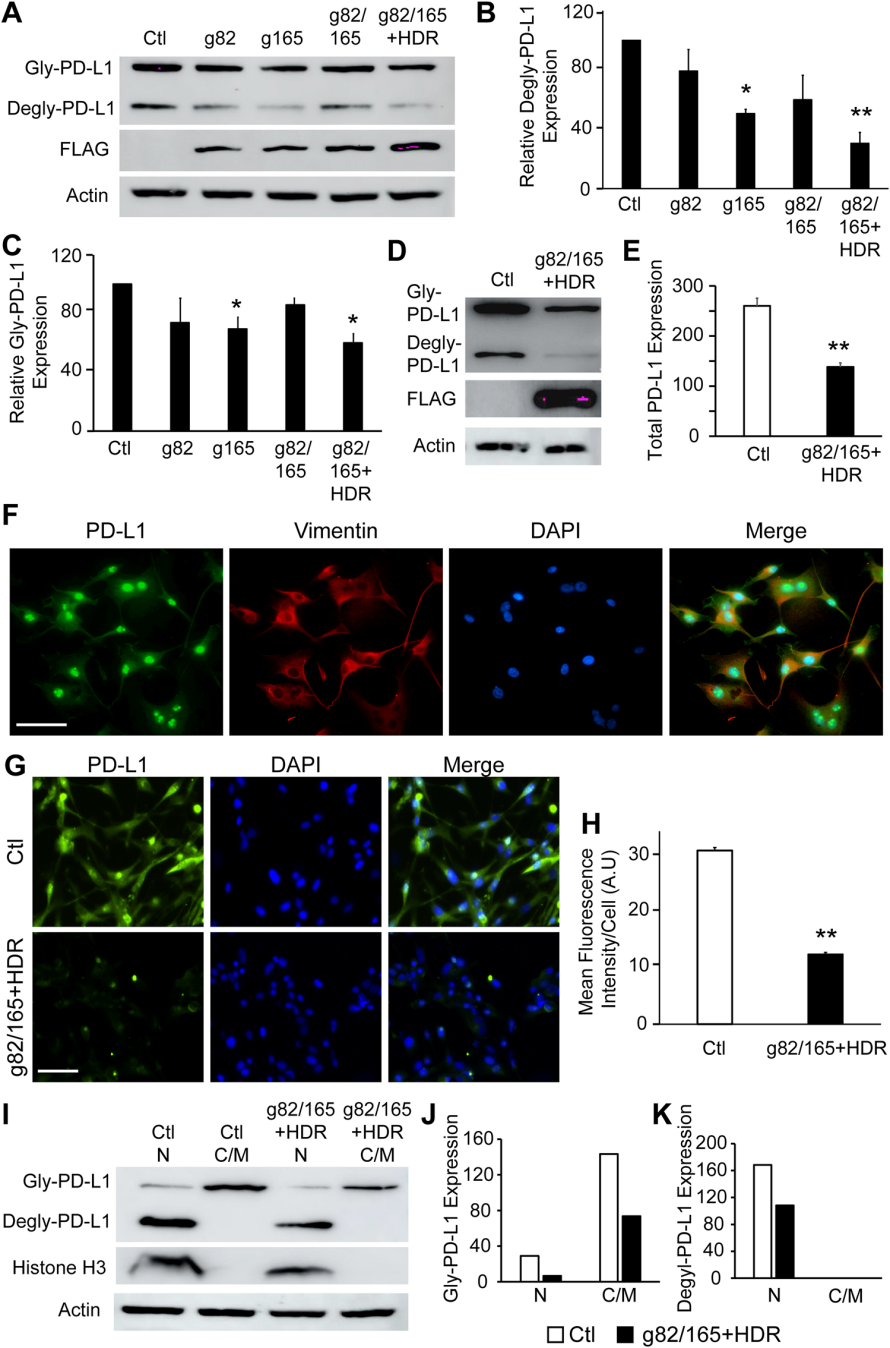
PD-L1 knockout by CRISPR/Cas9 plasmids in U87 cells. (**A**) Western blot analysis of PD-L1 protein levels. U87 cells transfected with the CRISPR/Cas9 plasmids were analyzed and quantified to determine PD-L1 protein knockout efficiency of both (**B**) deglycosylated and (**C**) glycosylated PD-L1. The strongest PD-L1 knockout was detected in Cas9-g82/165 + HDR treated U87 cells. (**D** and **E**) PD-L1 knockout by Cas9-g82/165 + HDR was further confirmed by western blot analysis in human breast cancer MDA MB 231 cells. (**F**) PD-L1 localization in U87 cells. U87 cells were stained for PD-L1, and PD-L1 expression (green) was examined on the membrane and cytoplasm using the structural protein vimentin (red), and the nucleus using DAPI (blue). Images suggests PD-L1 is expressed throughout the cell, with high expression seen in the nucleus. (**G**) Immunofluorescence labeling of PD-L1 in U87 cells treated with or without Cas9-g82/165 + HDR. (**H**) Quantification of the mean fluorescence intensity of individual cells suggests that PD-L1 was significantly reduced in Cas9-g82/165 + HDR treated U87 cells. Control cell # = 266, Cas9-g82/165 + HDR treated cell # = 253. (**I**) Western blot analysis of nuclear and membrane/cytoplasm fractions of U87 cells confirms PD-L1 expression in the cytoplasm and nucleus. Histone H3 was used to identify the nuclear fractions. Cells treated with Cas9-g82/165 + HDR showed a significant decrease in PD-L1 in both the membrane and nuclear fractions. Quantification of (**J**) glycosylated and (**K**) deglycosylated PD-L1 in the nuclear and membrane fractions of U87 cells treated with or without Cas9-g82/165 + HDR. (**D**–**E**) and (**I**–**K**) represent a single experiment. All other experiments were performed in triplicate and results were normalized for direct comparisons. Data represents the mean ± SEM. Statistical significance was evaluated with either a one-way ANOVA followed by Tukey’s post-hoc analysis (**B**, **C**), or a t-test (**E** and **H**). *p < 0.05, **p < 0.01. Scale bar = 50 µm.

To examine PD-L1 protein knockout efficiency, U87 cells were treated with Cas9-g82, Cas9-g165, Cas9-g82/165, and Cas9-g82/165 + HDR, and PD-L1 protein levels were analyzed by western blot analysis. Protein extracts were examined with antibodies against PD-L1 and the housekeeping gene actin to quantify PD-L1 protein levels (Fig. 3A, Supplemental Fig. 3). Quantitative western blot analysis indicated a reduction of deglycosylated and glycosylated PD-L1 protein levels in Cas9-g82/165 + HDR and Cas9-g165 treated U87 cells compared to the control (Fig. 3B, C). However, the greatest PD-L1 knockout was obtained in Cas9-g82/165 + HDR treated cultures that show a 64% total reduction of PD-L1 protein levels. Knockout of PD-L1 by Cas9-g82/165 + HDR was also confirmed in a human breast cancer cell line MDA MB 231) (Fig. 3D, E, Supplemental Fig. 4), suggesting Cas9-g82/165 + HDR was capable of targeting human PD-L1.

**Figure 4:**
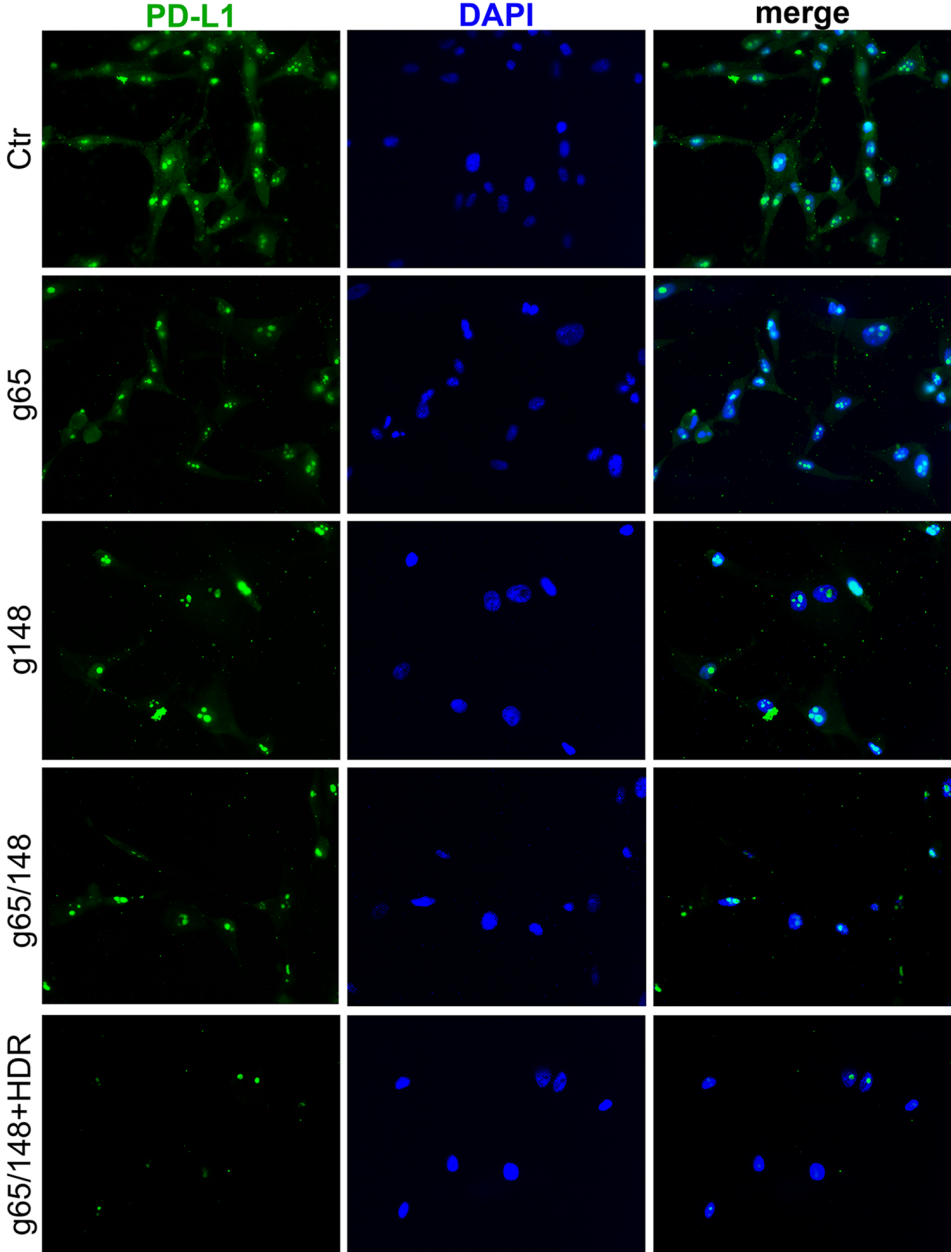
Immunostaining of PD-L1. The cytoplasmic and nuclear PD-L1 were stained with antibody against PD-L1 (green). The nuclei was labeled with DAPI (blue). Dual immunofluorescence labeling of PD-L1 and DAPI in U87 cells treated to Cas9-g82, Cas9-g165, Cas9-g82/165, and Cas9-g82/165 + HDR.The untreated U87 cells served as control. Notable reduction of both cytoplasmic and nuclear PD-L1 was seen in U87 cells treated to Cas9-g82/165 + HDR.

PD-L1 is expressed on the membrane, nuclei and cytoplasm of human U87 cells (Fig. 3F). To examine the cellular distribution of PD-L1 after PD-L1 knockout by Cas9-g82/165 + HDR, we performed an immunofluorescence assay (Fig. 3G). U87 cells were stained with antibody against PD-L1 (green), and the nucleus was labeled with DAPI (blue). U87 cells transfected with lipofectamine only served as the control. High expression of PD-L1 was seen in both the cytoplasm and the nucleus (Fig. 3G, green). In contrast, Cas9-g82/165 + HDR treated U87 cells had significantly reduced PD-L1 expression in both the cytoplasm and nucleus. GFP + expression was quantified by analyzing the mean fluorescence intensity of individual cells (Fig. 3H). A significant reduction of PD-L1 fluorescence intensity was obtained in Cas9-g82/165 + HDR treated U87 cells (Fig. 3H, *p* < 0.01). To confirm the loss of PD-L1 in the nucleus, cytoplasm, and membrane, U87 cells were transfected with or without Cas9-g82/165 + HDR, and the cells were lysed to obtain nuclear and cytoplasmic/membrane fractions. These protein extracts were stained for PD-L1, histone H3, and FLAG (Fig. 3I, Supplemental Fig. 4). Histone H3 was used to identify the nuclear fraction (Ctl N). The cytoplasmic/membrane fraction (Ctl C/M) does not express histone H3. Western blot analysis revealed glycosylated PD-L1 is expressed in both the nuclei and the cytoplasmic/membrane fractions, respectively. In contrast, deglycosylated PD-L1 was only detected in the nuclear fraction as indicated by the presence of histone H3 (nuclear). Further, PD-L1 knockout by Cas9-g82/165 + HDR lead to the reduction of PD-L1 in both nuclear (Cas9-g82/165 + HDR N) and cytoplasmic /membrane (Cas9-g82/165 + HDR C/M) fractions (Fig. 3I). Quantification of glycosylated and deglycosylated PD-L1 (Fig. 3J, K) in the nuclear and membrane fractions of cells treated with or without Cas9-g82/165 + HDR. Further, fluorescence labeling of PD-L1 (Fig. 4, green) and the nuclei with DAPI (Fig. 4, blue) confirmed that both nuclear and cytoplasmic PD-L1 were notably reduced in Cas9-g82/165 + HDR (Fig. 4). These results support the conclusion that Cas9-g82/165 + HDR can specifically and efficiently knockout PD-L1 in human U87 glioblastoma cells.

### PD-L1 deletion inhibits U87 proliferation

**Figure 5:**
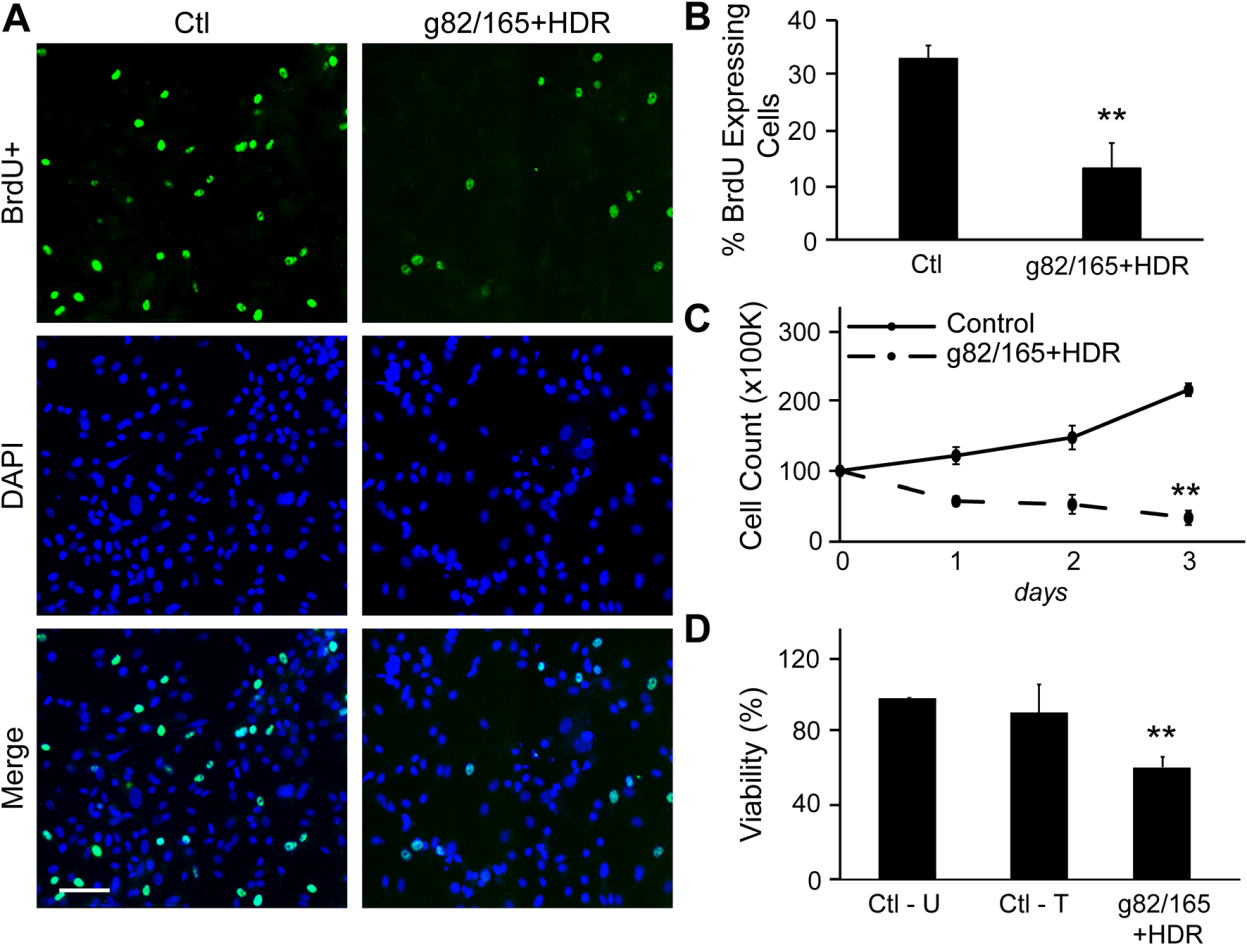
PD-L1 knockout slows U87 tumor cell proliferation and growth. (**A**) BrdU staining was performed to analyze cell proliferation. Images suggest that U87 cells treated with Cas9-g82/165 + HDR proliferate at a slower rate compared to control cells. (**B**) Quantification of A. (**C**) Quantification of the growth rate of U87 cells transfected with or without Cas9-g82/165 + HDR. Cells were counted over 3 days. Images revealed that PD-L1 deletion inhibited U87 cell growth compared to the control. (**D**) An MTT assay was performed to examine U87 cell viability after treatment with Cas9-g82/165 + HDR for 2 days. Untreated cells (Ctl-U) and cells treated with lipofectamine only (Ctl-T) served as the controls. U87 cells treated with Cas9-g82/165 and Cas9-g82/165 + HDR displayed greater toxicity compared to the control and cells treated with the individual sgRNAs. All experiments were performed in triplicate and results were normalized for direct comparisons. The data represent the mean ± SEM, and statistical significance was evaluated with either a t-test (**B** and **C**) or a one-way ANOVA followed by Tukey’s post-hoc analysis (**D**). **p < 0.01. Scale bar = 100 µm.

The effects of PD-L1 knockout by Cas9-g82/165 + HDR on cell proliferation and growth were examined in U87 cells. BrdU immunostaining was performed to determine U87 cell proliferation with or without Cas9-g82/165 + HDR treatment. Imaging BrdU staining (Fig. 5A, green) revealed that deletion of PD-L1 by Cas9-g82/165 + HDR inhibited U87 cell proliferation. Quantitative imaging showed that PD-L1 deletion statistically reduced BrdU + proliferating U87 cells (Fig. 5B) compared to the control. Further, U87 cells treated with or without Cas9-g82/165 + HDR were counted on days 1, 2 and 3 to determine the growth rate. The total number of U87 cells was significantly decreased when PD-L1 was knocked out compared to the control (Fig. 5C, Supplemental Fig. 5). Finally, an MTT assay was used to determine the viability of U87 cells after PD-L1 deletion (Fig. 5D). U87 cells were transfected with either lipofectamine (Ctl—T) or Cas9-g82/165 + HDR. Untreated cells served as an additional control (Ctl—U). A significant decrease of cell viability was detected in U87 cells treated with Cas9-g82/165 + HDR, that was not caused by lipofectamine transfection. Together, these data suggest that PD-L1 deletion by Cas9-g82/165 + HDR alters GBM growth and proliferation pathways to decrease U87 cell viability.

### PD-L1 deletion prevents U87 cell invasion and migration

**Figure 6:**
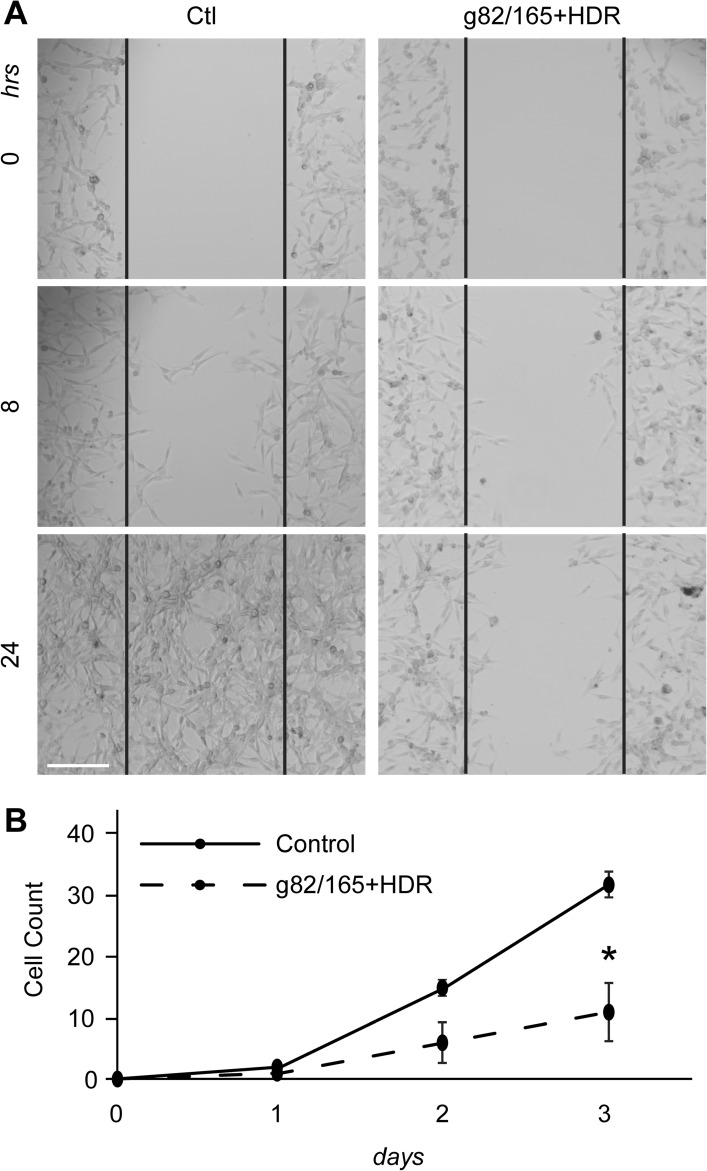
PD-L1 knockout inhibits U87 cell invasion and migration. (**A**) A scratch assay was performed to examine U87 cell invasion after PD-L1 knockout by Cas9-g82/165 + HDR. Invasion of U87 cells into the scratched area was analyzed within the black lines. U87 cells treated with Cas9-g82/165 + HDR were less invasive compared to the control. (**B**) A migration assay using a 3 µm insert was used to analyze migratory potential. Cells were plated in the insert, and their migration to the plate surface was monitored over three days. The number of cells that migrated were counted, and results were normalized across 3 experiments. The data suggests Cas9-g82/165 + HDR slowed migration of U87 cells compared to control. The data represent the mean ± SEM and statistical significance was evaluated with a t-test (**B**). *p < 0.05. Scale bar = 200 µm.

U87 cell invasion and migration with or without Cas9-g82/165 + HDR treatment was evaluated using a cell scratch assay and a two-layer insert culture system. For the invasion assay, U87 cells were plated on a 12 well plate and treated with Cas9-g82/165 + HDR. Cells were scratched 24 h post-treatment. The invasion of U87 cells into the scratched area was examined over 24 h (Fig. 6). PD-L1 deletion by Cas9-g82/165 + HDR prevented U87 cell invasion compared to the control (Fig. 6A), as seen at 8 and 24 h.

A two-layer insert culture system was used to examine PD-L1 knockout-mediated inhibition of U87 cell migration. U87 cells were plated on an insert (top layer) with a 3 µm pore and treated with Cas9-g82/165 + HDR. U87 cells migrating from the top layer of the insert to the bottom layer of the plate was examined by microscopy. The number of U87 cells that migrated to the bottom layer were counted on days 1, 2 and 3. PD-L1 knockout in U87 cells resulted in a significant reduction in cell migration (Fig. 6B). Together, these data suggests deletion of PD-L1 inhibits U87 cell invasion and migration.

### PD-L1 knockout in U87 cells regulates TAM polarization

**Figure 7:**
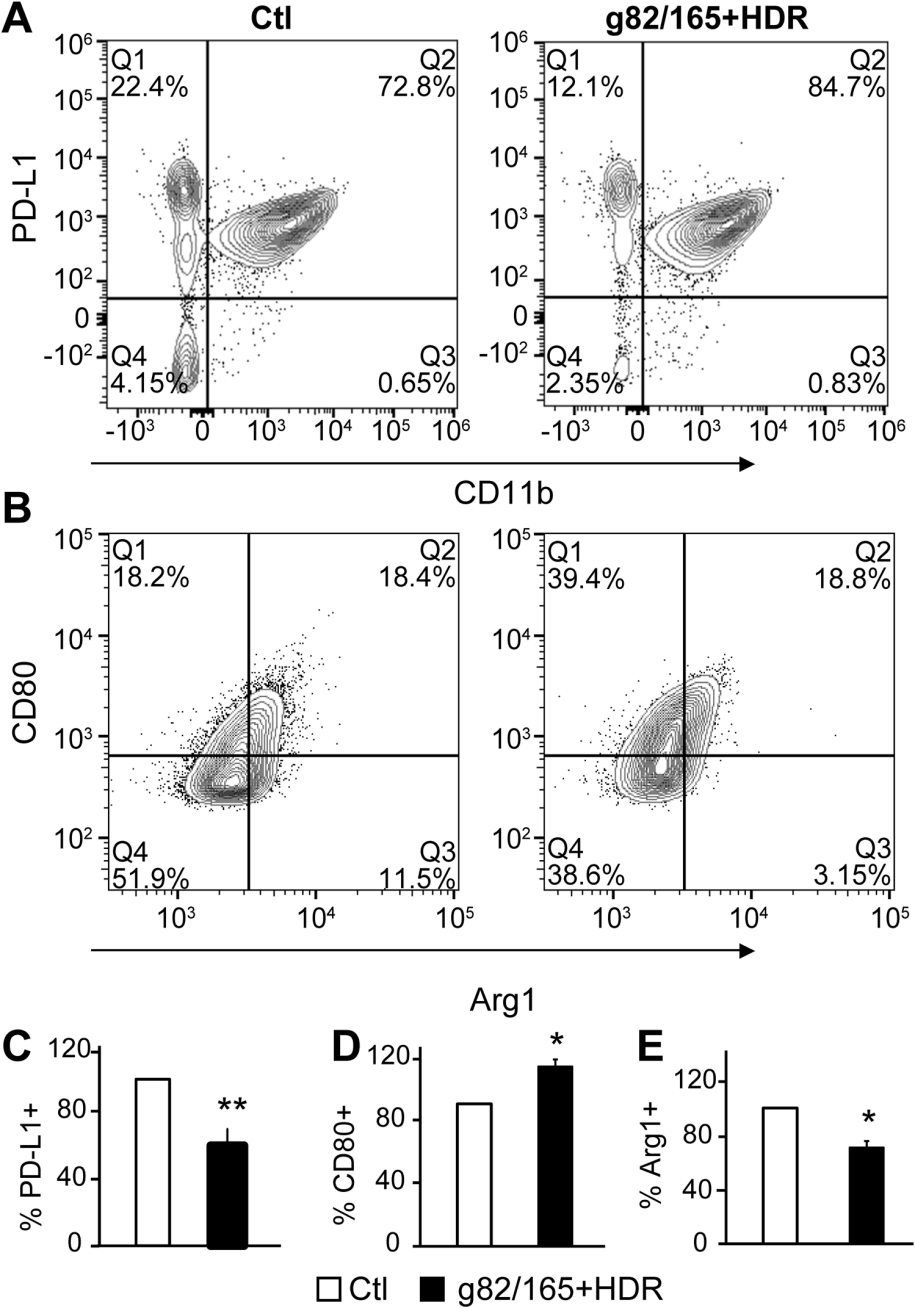
PD-L1 knockout in U87 cells polarizes human TAMs toward an M1 phenotype. U87 cells were treated with Cas9-g82/165 + HDR for 24 h, then human macrophages were added allowing TAM differentiation for 48 h. The co-cultures were collected and analyzed by flow cytometry. Contour plots of (**A**) CD11b and PD-L1, and (**B**) Arginase I and CD80 subpopulations are shown for Cas9-g82/165 + HDR treated cultures and the control. (**C**) PD-L1 knockout in U87 cells was confirmed by gating for CD45-CD11b- cells and examining PD-L1 + cells. Co-cultivation of TAMs and U87 cells treated with Cas9-g82/165 + HDR repolarized M2 TAMs into (**D**) CD80 + M1 TAMs. In contrast, PD-L1 knockout in U87 cells leads to (**E**) Arginase I + M2 depletion. All experiments were performed in triplicate and results were normalized for direct comparisons. Data represents the mean ± SEM. Statistical significance was evaluated with a t-test. *p < 0.05, **p < 0.01.

Microglia are the resident macrophages of the brain and are the only immune cell type found in this immune privileged organ. Therefore, to investigate the impact of PD-L1 knockout by Cas9-g82/165 + HDR in a GBM environment, we developed an in vitro GBM model by co-cultivating U87 cells with either a human macrophage cell line or primary mouse macrophages derived from mouse bone marrow (BMMs)**.** Co-cultivation of macrophages with tumor cells commonly polarizes TAMs toward an M2 phenotype. We divided the co-cultivation system into two groups. One group contained U87 cells pre-treated with Cas9-g82/165 + HDR for 24 h to knockout PD-L1, then macrophages were added 24 h later and were co-cultured for 2 days, allowing TAM differentiation. The second group contained untreated U87 cells co-cultured with macrophages to serve as the control. TAM polarization by U87 cells with or without PD-L1 knockout was assessed by flow cytometry. The gating strategy for human macrophages and mouse BMM are illustrated in Supplemental Figs. 6 and 7. Gating of human CD45-CD11b- cell populations was used to identify U87 cells, while CD45 + CD11b + gated cells were used to identify human TAMs. Representative plots show subsets of CD45-CD11b-PD-L1 + U87 cells co-cultured with human macrophages (Fig. 7A). Cas9-g82/165 + HDR significantly reduced intracellular PD-L1 + U87 cells compared to the control (Fig. 7C, (*p* < 0.05)*.* This confirmed Cas9-g82/165 + HDR was effective at PD-L1 knockout. Human CD45 + CD11b + TAMs were further gated for Arginase I (M2 marker) and CD80 (M1 marker) to identify TAM polarization (Fig. 7B). The CD80 + M1 marker was upregulated in co-cultures with PD-L1 deleted U87 cells (Fig. 7D, *p* < 0.05). In contrast, a significant reduction of Arginase I + M2 TAMs (Fig. 7E, *p* < 0.05) was detected in the co-cultures with Cas9-g82/165 + HDR pre-treated U87 cells. In addition, primary mouse BMM polarization by U87 cells with or without Cas9-g82/165 + HDR was examined by analyzing PD-L1 + U87 cells, and Ly6C + , Ly6G + , CD206 + , and CD80 + TAMs (Supplemental Fig. 8A–C). Deletion of PD-L1 + in U87 cells (Supplemental Fig. 8D) correlated with an increase of Ly6C + and CD80 + M1 TAMs (Supplemental Fig. 8E, F, *p* < 0.05) and a decrease of CD206 + M2 TAMs (Supplemental Fig. 8G, *p* < 0.01). This M2/M1 shift indicated that the loss of PD-L1 in U87 cells can up-regulate M1 TAMs and down-regulate M2 populations in an in vitro GBM environment.

### Changes in TAM function by PD-L1 knockout in U87 cells

**Figure 8:**
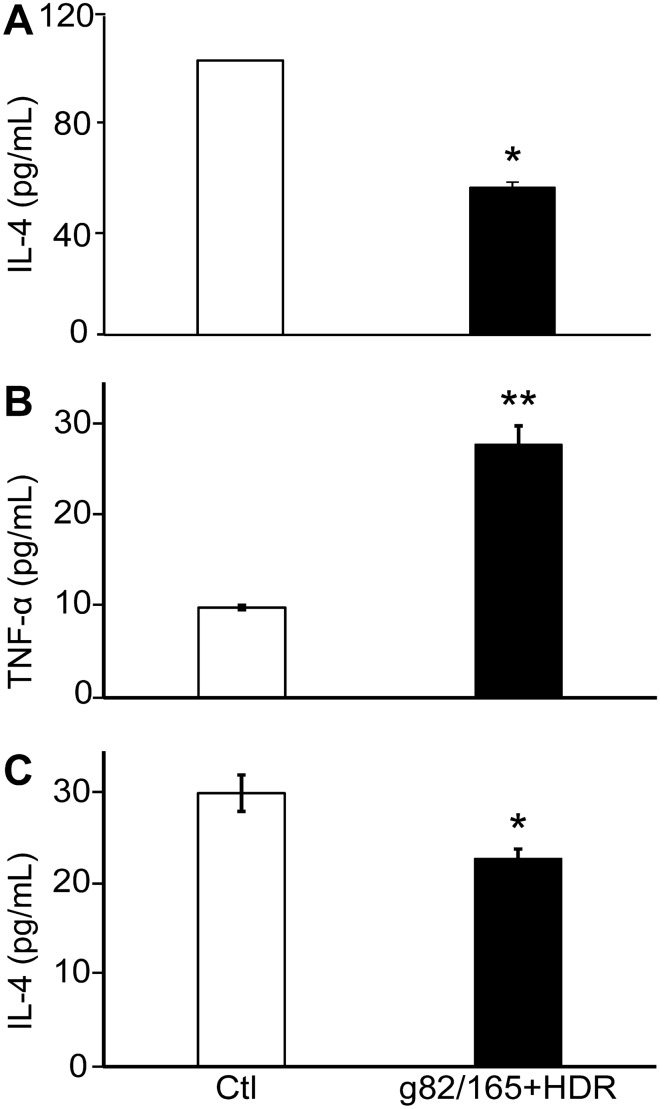
Functional polarization of TAMs by PD-L1 knockout in U87 cells. U87 cells were treated with Cas9-g82/165 + HDR for 24 h, then human macrophages were added allowing TAM differentiation for 48 h. An ELISA assay was used to detect (**A**) human IL-4 secretions in the medium from co-cultured human macrophages and U87 cells with or without Cas9-g82/165 + HDR. In parallel, mouse BMMs co-cultured with U87 cells were investigated to examine PD-L1 knockout on mouse (**B**) TNF-α and (**C**) IL-4 secretions**.** PD-L1 deletion in U87 cells significantly upregulated TNF-α levels and reduced IL-4 secretion compared to the control. All experiments were performed in triplicate and results were normalized for direct comparisons. Data represents the mean ± SEM. Statistical significance was evaluated with a t-test. *p < 0.05, **p < 0.01.

Cytokine secretions were used to detect functional polarization of TAMs by PD-L1 knockout in the human U87/macrophage co-cultivation system. Soluble IL-4 (M2 marker) secretions in the culture medium were determined by ELISA. The secretion of IL-4 was markedly suppressed (Fig. 8A) in co-cultures of human macrophages and U87 cells with or without Cas9-g82/165 + HDR at day 3. This was further confirmed using the mouse BMM co-cultivation system. An increase of TNF-α (M1 marker) (Fig. 8B, *p* < 0.01) and a decrease of IL-4 (M2 marker) (Fig. 8C, *p* < 0.05) were seen in the Cas9-g82/165 + HDR treated group compared to the control. Consistently, the reduction of soluble IL-4 in the medium correlated to a decrease of Arginase I + and CD206 + M2 TAMs (Fig. 7). Moreover, a high level of TNF-α from mouse BMMs co-cultured with Cas9-g82/165 + HDR pre-treated U87 cells compliments the elevation of Ly6C +, and CD80 + M1 TAMs (Supplemental Fig. 8). These findings strongly supported that PD-L1 knockout in U87 cells can directly regulate TAM functional polarization. Taken together, our data indicates that genomic deletion of PD-L1 in U87 cells can alter the tumor environment, leading to a potential anti-GBM strategy.

### PD-L1 knockout in vivo regulates TAM polarization

**Figure 9:**
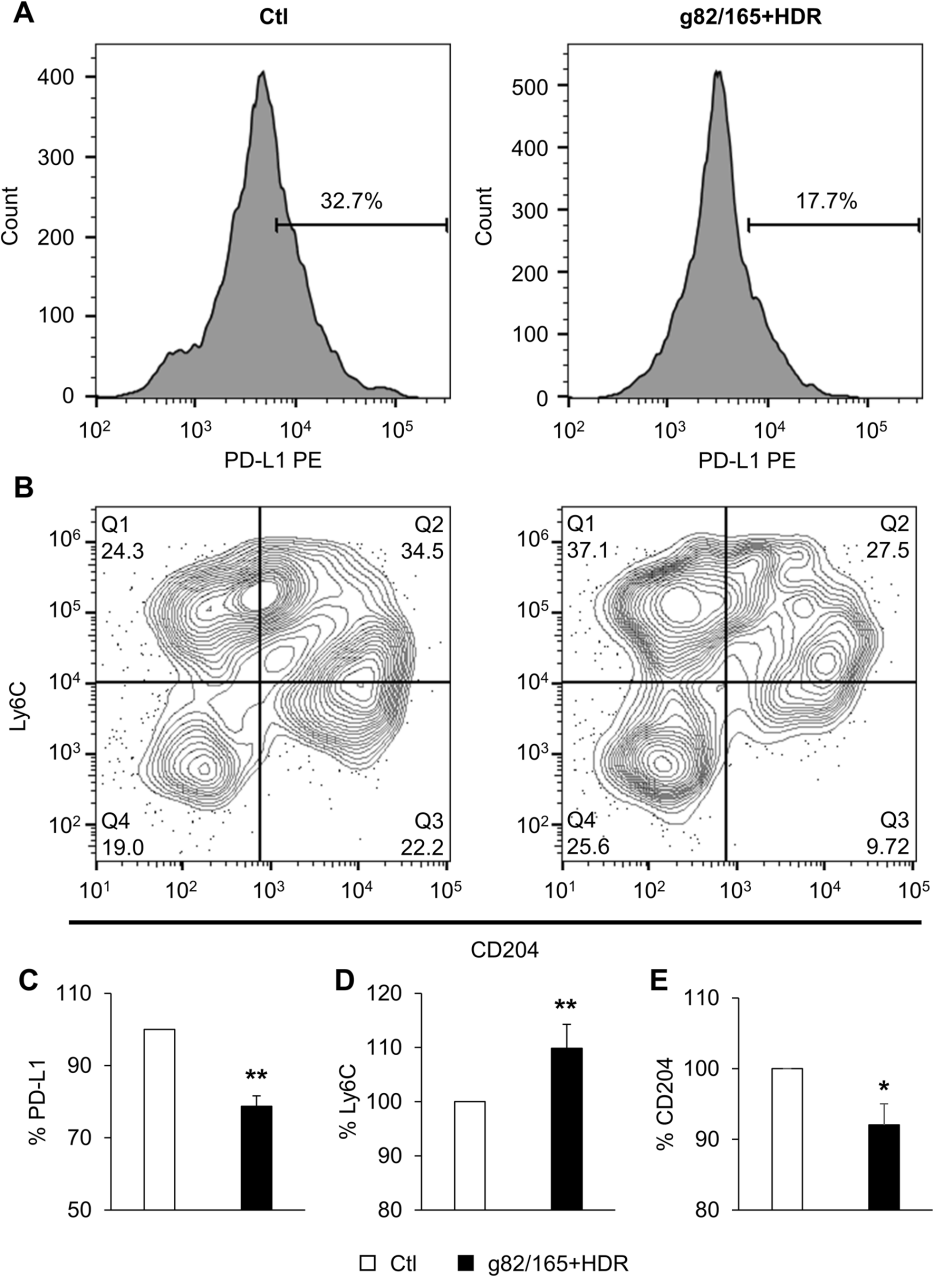
PD-L1 knockout in vivo polarizes TAMs to M1 in the spleen. Balb/C mice were injected through the tail vein with either nanoparticles or nanoparticles loaded with Cas9-g82/165 + HDR every three days for 9 days, then mice were sacrificed to examine macrophage polarization in the spleen by flow cytometry. Histograms of (**A**) PD-L1 and contour plots for (**B**) Ly6C and CD204 subpopulations for the spleen are shown for Cas9-g82/165 + HDR treated mice and the control. (**C**) Mice treated with Cas9-g82/165 + HDR showed a significant reduction of PD-L1 in spleen myeloid cells, indicating successful knockout by Cas9-g82/165 + HDR. Further, PD-L1 knockout polarized M2 TAMs into (**D**) Ly6C + M1 TAMs. This was further confirmed by examining (**E**) CD204 + M2 TAMs, which showed a significant decrease in spleen macrophages. n = 9 mice per condition over three experiments. The data was normalized for direct comparisons and represents the mean ± SEM. Statistical significance was evaluated with a t-test. *p < 0.05, **p < 0.01.

In order to test the effectiveness of PD-L1 knockout in vivo, we injected mice with nanoparticles loaded with Cas9-g70/166 + HDR (mouse guides and HDR template) via the tail vein. This nanoparticle has been shown to be effective at transfecting cells in vitro and in vivo^46,47^. Mice injected with nanoparticles only served as the control (Ctl). Mice were sacrificed 9 days post the initial injection, and the spleen was harvested and analyzed by flow cytometry. PD-L1 knockout was analyzed on splenic cells following the gating strategy illustrated in Supplemental Fig. 9. Representative plots show the reduction in PD-L1 + splenic cells (Fig. 9A, C) by Cas9-g82/165 + HDR**.** Splenic myeloid cells were further gated for CD45 + CD11b + F4/80 + macrophages, which were further gated for F4/80 + Ly6C + M1 and F4/80 + CD204 + M2 macrophages. Importantly, a significant increase of F4/80 + Ly6C + M1 macrophages was obtained in mice treated with Cas9-g70/166 + HDR-NPs compared to control mice (Fig. 9B, D, *p* < 0.01). Further, a significant decrease in F4/80 + CD204 + M2 macrophages was observed in Cas9-g82/165 + HDR treated mice (Fig. 9B, E, *p* < 0.05). These data further support the evidence of M2 polarization by PD-L1 knockout after treating mice with Cas9-g70/166 + HDR.

## Discussion

GBM is a devastating disease with an average patient survival expectancy of 12–18 months post diagnosis. PD-L1 has shown promise as a therapeutic target for GBM^48,49^, but currently no efficient therapies exist to target PD-L1 in GBM. We developed a CRISPR/Cas9 gene-editing strategy with two-sgRNAs and an HDR template to disrupt PD-L1 function in human GBM U87 cells. sgRNAs g82 and g165 were successfully cloned into a single plasmid using the multiplex CRISPR/Cas9 assembly system^44^. The Cas9-g82/165 plasmid was co-transfected with a synthetic HDR template that enhanced PD-L1 knockout compared to individual sgRNA’s and dual-sgRNAs without the HDR template. We found the loss of PD-L1 in U87 GBM cells led to the inhibition of cell growth, proliferation, and migration, and it also led to the polarization of TAMs from M2 to M1. Using mouse guides g70 and g166 to target mouse PD-L1 in vivo also knocked out PD-L1 and caused polarization of macrophages from M2 to M1.

The CRISPR/Cas9 system has become the gold standard for precise gene editing and has great potential for the treatment of many diseases and disorders^45^. CRISPR/Cas9 gene editing is a remarkable tool for modifying genes in vitro and in vivo^50–52^. However, off-target effects and gene-editing specificity are critical challenges for biomedical research and clinical applications. Off-target effects result from a sgRNA recognizing an unintended DNA target that leads to cutting outside of the target gene^36–39^. In addition, cuts introduced by Cas9 nuclease can be repaired by NHEJ or HDR pathways differently in each cell. This leads to inefficient gene-editing that can result in a mosaic of outcomes, including silent mutations. New breakthroughs in CRISPR/Cas9 gene editing have allowed researchers to manipulate this system to induce the desired mutation. For example, using an HDR template allows for the incorporation of an in-frame stop codon, permitting specific knockout of the target gene in all cells^53–55^. Our designed dual-sgRNA CRISPR-Cas9 plasmid generates two cuts (adjacent to each other) on the genomic DNA, preventing off-target effects and improving genomic editing specificity and therapeutic accuracy. Co-transfecting this plasmid with the HDR template that incorporates an in-frame stop codon improved PD-L1 knockout efficiency. This strategy ensures a null mutation occurs rather than a hyper-, hypo-, neomorphic, or silent mutation.

Knockout of PD-L1 by Cas9-g82/165 + HDR demonstrates that the dual-sgRNA plasmid with the HDR template can edit *PD-L1* at the genomic level, which leads to inhibition of protein production. While all constructs show some degree of gene-editing in human GBM U87 cells, Cas9-g82/165 + HDR provided the strongest PD-L1 knockout. This dual-sgRNA strategy with an HDR template greatly enhances CRISPR/Cas9 gene-editing efficiency to knockout PD-L1 in U87 cells, and was confirmed to knockout PD-L1 in human breasts cancer cells.

It is well-known that PD-L1 is an immune checkpoint regulator. Membrane-bound PD-L1 contributes to tumor-induced immune resistance by binding to PD-1^56,57^. However, intracellular PD-L1 has been poorly studied. During tumor progression, high PD-L1 expression in tumor cells is closely associated with tumor growth and migration^58–61^. Studies suggests this may occur by activating WIP and beta catenin signaling pathways, although more work is needed to understand how PD-L1 activates these pathways^62^. Numerous studies have focused on inhibition of membrane-bound PD-L1 to develop anti-cancer immunotherapy. The disadvantage of targeting cell surface PD-L1 is that intracellular PD-L1 promotes tumor growth and migration, and can translocate to the cell surface to continue inducing immunosuppression^63^. Our study showed that PD-L1 is highly expressed in the cytoplasm and nucleus of U87 cells. In particular, Cas9-g82/165 + HDR significantly reduced cytoplasmic and nuclear PD-L1, while also being associated with inhibition of U87 cell proliferation, growth, invasion and migration. These results are consistent with other studies that show PD-L1 is important for tumor growth and progression^49,62–64^.

Further, we found that PD-L1 knockout in U87 cells polarized TAMs from M2 into M1. The effects of PD-L1 knockout could be a direct anti-GBM therapeutic strategy over commonly reported PD-1/PD-L1 inhibitory immunotherapy. Our data suggested that Cas9-g82/165 + HDR treated U87 cells caused a reduction of M2 TAM polarization and an increase in M1 TAM polarization in an in vitro GBM environment. The in vitro co-cultivation system also revealed PD-L1 deletion can suppress M2 IL-4 secretion and upregulate M1 TNF-α secretion. Therefore, our findings indicate PD-L1 deletion from U87 cells promotes suppressive M2 TAM polarization into M1 immune activation, contributing to anti-tumor immune therapy on GBM. This was also found to be true using mouse Cas9-g70/166 + HDR to knockout PD-L1, which demonstrated PD-L1 knockout can polarize macrophages in vivo*.* The benefit of the Cas9 dual-sgRNA plus HDR system is that genomic deletion of PD-L1 inhibits both cell surface and intracellular protein expression, and disrupts its membrane replenishment. Cas9-g82/165 + HDR-mediated PD-L1 deletion could lead to an effective anti-GBM effect over anti-PD-L1 therapy.

Inhibition of PD-L1 has shown great promise for the treatment of several cancers. However, these strategies have not been successful in the treatment of GBM. Our work lays the foundation for using two-sgRNAs and an HDR template to enhance PD-L1 knockout efficiency, specificity, and accuracy. The loss of PD-L1 in U87 cells prevents tumor growth, proliferation, and migration, inhibiting GBM immune resistance. This dual-sgRNA CRISPR/Cas9 gene-editing therapeutic strategy is therefore a novel method for treating GBM. This study is the first of its kind to employ a CRISPR/Cas9 strategy using dual-sgRNAs and an HDR template to knockout PD-L1 in human GBM tumor cells. This dual-sgRNA CRISPR/Cas9 and HDR strategy has great implications for translation into the clinical setting for treating GBM.

## Methods

### Design and construction of sgRNA and dual-sgRNA containing CRISPR/Cas9 plasmids

Human and mouse PD-L1 guides were designed with Integrated DNA Technologies (IDT, Coralville, Iowa) gRNA design tool (https://www.idtdna.com/site/order/designtool/index/CRISPR_SEQUENCE), and were further modified to include BbsI cut sites (Table 1). Both mouse guides recognize the forward and reverse strands of mouse PD-L1, while human guide 82 (g82) and guide 165 (g165) recognize the forward strand of human PD-L1. 100 µM of the forward and reverse primers were annealed together in 10X annealing buffer (100 mM Tris, pH 7.5, 500 mM NaCl, 10 mM EDTA) for 5 min at 95 °C, then cooled to room temperature for 1 h. Annealed primers were cloned into the multiplex CRISPR/Cas9 assembly system^44^ (Addgene, Watertown, MA) and into pSpCas9(BB)-2A-GFP (PX458)^45^ (Addgene, Watertown, MA). Positive clones were identified by Sanger sequencing (Genewiz, South Plainfield, NJ). Plasmid DNA was prepared with the Qiagen Plasmid Midi Kit (Qiagen, Hilden, Germany) and quantified with a Nanodrop 1000 spectrophotometer (Thermo Fisher Scientific, Waltham, MA).

**Table 1:** Primer sequences used in this study.

Purpose	Primer name	Primer sequence
Cloning	Hu PD-L1 Cas9-g82 Forward	5Phos—CAC CGG GTT CCC AAG GAC CTA TAT G
Cloning	Hu PD-L1 Cas9-g82 Reverse	5Phos—AAA CCA TAT AGG TCC TTG GGA ACC C
Cloning	Hu PD-L1 Cas9-g165 Forward	5Phos—CAC CGT GGC TGC ACT AAT TGT CTA T
Cloning	Hu PD-L1 Cas9-g165 Reverse	5Phos—AAA CAT AGA CAA TTA GTG CAG CCA C
Transfection	Hu PD-L1 Repair Template	TGG CAT TTG CTG AAC GCA TTT ACT GTC ACG GTT CCC AAG GAC CTA TAA TAT TGA GAA ATG GAG GAT AAG AAC ATT ATT CAA TTT GTG CAT GGA G
Cloning	Ms PD-L1 Cas9-g70 Forward	5Phos—CAC CGG TTT ACT ATC ACG GCT CCA A
Cloning	Ms PD-L1 Cas9-g70 Reverse	5Phos—AAA CTT GGA GCC GTG ATA GTA AAC C
Cloning	Ms PD-L1 Cas9-g166 Forward	5Phos—CAC CGA GTA CAC CAC TAA CGC AAG C
Cloning	Ms PD-L1 Cas9-g166 Reverse	5Phos—AAA CGC TTG CGT TAG TGG TGT ACT C
Transfection	Ms PD-L1 Repair Template	TTC ACA GCC TGC TGT CAC TTG CTA CGG GCG TTT ACT ATC ACG GCT TAA GCG TTA GTG GTG TAC TGG GAA AAG GAA GAT GAG CAA GTG ATT C
T7E1	Hu PD-L1 Screen Forward	ACA TAA CCG ACC AGA TAA AGT GAT
T7E1	Hu PD-L1 Screen Reverse	AGT CTT CAA CAC TTG GAA TAT GTT T

The human and mouse repair templates were designed and analyzed with IDT’s oligo analyzer tool. Both repair templates include an in-frame stop codon after the first CRISPR/Cas9 cut site. To prevent the destruction of the human repair template at the g165 cut site, a single base pair mutation at base pair 171 (G → A; Table 1) was incorporated. This destroys the PAM sequence for the second guide to prevent Cas9 from cutting the repair template. All primers were ordered from IDT (Coralville, Iowa).

### Cell culture and transfection

U87 cells (ATCC, Manassas, VA) and MDA MB 231 cells (gift from Gadad Lab) were cultured in growth media consisting of Dulbecco’s-Modified Eagle Medium (DMEM, Sigma-Aldrich, St Louis, MO) supplemented with 10% fetal bovine serum (FBS; Sigma-Aldrich, St Louis, MO) and 1% penicillin–streptomycin (Thermo Fisher Scientific, Waltham, MA) at 37 °C with 5% CO2. Cells were transfected with 1.5ug CRISPR/Cas9 plasmid DNA per mL of media 24 h later using 2.5uL Lipofectamine 3000 per mL (Thermo Fisher Scientific, Waltham, MA). For cells treated with Cas9-g82/165 + HDR, the single stranded synthetic HDR template was co-transfected with g82/165 plasmid DNA to obtain a final concentration of 12.5 µM. Varying concentrations of the CRISPR/Cas9 constructs and HDR template were tested to experimentally determine the strongest knockout of PD-L1. Cells treated with lipofectamine only served as the control in all experiments.

Human SC cells (ATCC, Manassas, VA) were cultured in growth media consisting of Iscove’s Modified Dulbecco’s Medium (ATCC, Manassas, VA) supplemented with 0.05 mM 2-mercaptoethanol (Fisher Scientific, Hampton, NH), 0.1 mM hypoxanthine (Fisher Scientific, Hampton, NH), 0.016 mM thymidine (Fisher Scientific, Hampton, NH), 10% fetal bovine serum (FBS; Sigma-Aldrich, St Louis, MO) and 1% penicillin–streptomycin (Thermo Fisher Scientific, Waltham, MA) at 37 °C with 5% CO_2_.

Mouse bone marrow derived macrophage were obtained from femur of Balb/C mouse (male 5–6 weeks). The collected single-cell suspensions were cultured in Dulbecco’s-Modified Eagle Medium (DMEM, Sigma-Aldrich, St Louis, MO) supplemented with 10% fetal bovine serum (FBS; Sigma-Aldrich, St Louis, MO), 1% penicillin–streptomycin (Thermo Fisher Scientific, Waltham, MA), and 1000 U/ml highly purified recombinant macrophage colony stimulating factor (MCSF; R&D Systems, Minneapolis, MN) at 37 °C with 5% CO_2_ for 10–12 days allowing differentiation into macrophages.

### T7 endonuclease I (T7E1) assay

The Monarch Genomic DNA Purification Kit and the EnGen Mutation Detection Kit were purchased from New England Biolabs (Ipswich, MA), and the assays were carried out according to the manufacture’s protocols. Briefly, 150,000 cells were transfected with Cas9-g82, Cas9-g165, Cas9-g82/165, or Cas9-g82/165 + HDR using lipofectamine and were allowed to incubate for 2 days. Cells were harvested and the genomic DNA was isolated. The target site of interest was PCR amplified using primers ordered from IDT (Coralville, Iowa) with the Q5 Hot Start High Fidelity 2X Master Mix supplied with the EnGen Mutation Detection Kit. Primer sequences used in this study for cloning human (Hu) g82 and g165 and mouse (Ms) g70 and g166 into the multiplex CRISPR/Cas9 assembly system, the repair templates, and primers for the T7E1 assay are shown in Table 1. The PCR products were checked on an agarose gel, and then the samples were denatured for 5 min at 95 °C and allowed to anneal at room temperature for 1 h. Finally, the sample was digested with the T7 endonuclease 1 enzyme for 30 min at 37 °C followed by Proteinase K digestion for 5 min at 37 °C, and the resulting products were analyzed by agarose gel electrophoresis.

### Western blot

300,000 U87 cells were seeded in a 6 well plate and transfected with Cas9-g82, Cas9-g165, Cas9-g82/165, or Cas9-g82/165 + HDR the next day. 48 h post transfection, cells were washed twice in PBS and either lysed with RIPA buffer containing 1X protease inhibitor cocktail mix (Thermo Fisher Scientific, Waltham, MA), or cells were lysed with the nuclear extraction kit from Abcam (Cambridge, United Kingdom). Protein extracts were collected and quantified with the Pierce BCA protein assay kit (Thermo Fisher Scientific, Waltham, MA). 20 µg of protein was loaded onto a 12% SDS-PAGE gel (Bio-Rad Laboratories, Hercules, CA) and run for 1.5 h at 100 V. Samples were transferred onto a nitrocellulose membrane (Bio-Rad Laboratories, Hercules, CA) and checked by Ponceau staining (Sigma-Aldrich, St Louis, MO). Blots were washed in PBS to remove the staining, then blots were blocked in 3% milk (Bio-Rad Laboratories, Hercules, CA). Blots were stained with rabbit PD-L1 (1:500; Thermo Fisher Scientific, Waltham, MA), rabbit β-actin (1:500; Thermo Fisher Scientific, Waltham, MA), rabbit FLAG (1:500; Cell Signaling Technology, Danvers, MA) or rabbit Histone H3 (1:500; Novus Biologicals, Littleton, CO) in 3% milk overnight at 4 °C. Blots were washed in PBST (0.25% Tween-20 in PBS) and incubated in secondary antibody (rabbit IgG, HRP-linked antibody; 1:2000; Cell Signaling Technology, Danvers, MA) for 1 h. Samples were washed in PBST and imaged on an Amersham Imager 680 blot and gel imager (GE Healthcare, Chicago, IL). Quantification of blots was done with Image Pro Plus software (Media Cybernetics, Bethesda, MD), and data was normalized for direct comparisons using the formula $x^{\prime} = \frac{X - \mu }{\sigma }$, where X = the data value, µ = the mean of the data set, and σ = the standard deviation.

### Immunofluorescence

75,000 U87 cells were seeded on a #1.5 coverslip (Electron Microscopy Sciences, Hatfield, PA) in a 12 well plate and transfected the next day with either lipofectamine or lipofectamine + Cas9-g82/165 + HDR. 48 h later, cells were fixed in 4% paraformaldehyde, blocked with 2% NGS, and stained with rabbit PD-L1 overnight (1:500; Thermo Fisher Scientific, Waltham, MA) and mouse vimentin (1:500; Novus Biologicals, Littleton, CO). The next day, cells were washed in PBS 3 times and stained with goat anti-rabbit Alexa Fluor 488 (1:500; Thermo Fisher Scientific, Waltham, MA). Cells were washed 3 times in PBS and mounted onto slides using Fluoromount G with DAPI (Thermo Fisher Scientific, Waltham, MA). Slides were allowed to dry overnight and were imaged the following day using a Nikon Eclipse Ti fluorescence microscope. The mean fluorescence intensity was quantified using Image Pro Plus software, and the data was normalized for direct comparisons across experiments.

### MTT assay

Cell viability was assayed with the MTT cell proliferation kit according to the manufacturer’s instructions (Sigma-Aldrich, St Louis, MO). 35,000 U87 cells were plated on a 96-well flat bottom plate and transfected with Cas9-g82, Cas9-g165, Cas9-g82/165, or Cas9-g82/165 + HDR. Untreated cells and cells treated with lipofectamine only served as the controls. Samples were assayed on Day 2 using a FlexStation 3 Multi-Mode Microplate Reader (Molecular Devices, San Jose, CA) at 570 nm and 690 nm. Background absorbance taken at 690 nm was subtracted from the absorbance read at 570 nm. Samples were read in triplicate and the data collected was normalized to the controls for direct comparisons across experiments.

### Invasion and migration study

100,000 U87 cells were seeded in a 24 well plate and transfected with either lipofectamine only or Cas9-g82/165 + HDR 24 h later. 48 h post transfection, cells were scratched and their invasion into the scratched surface was imaged with an EVOS Digital Inverted Florescence Microscope (Thermo Fisher Scientific, Waltham, MA) at 0, 4, 8, and 24 h post scratching. To analyze cell migration, cells were seeded in a 3 µm insert placed in a 24 well plate, and cells were transfected with Cas9-g82/165 + HDR 24 h later. Migration from the insert to the plate was observed using a Nikon Eclipse Ti fluorescence microscope (Nikon Instruments, Melville, NY) over 3 days. The number of cells were counted, and the data was normalized for direct comparisons across experiments.

### Growth assay

100,000 U87 cells were plated on a 12 well plate and transfected with Cas9-g82/165 + HDR 24 h later. 24 h post transfection, control and treated cells were removed from the plate with trypsin and re-plated at 100,000 cells per well, 3 wells per condition. Growth rate was observed using a Nikon Eclipse Ti fluorescence microscope over 3 days, and by counting cells using a hemocytometer. The experiment was repeated in triplicate, and each time point was counted three times per experiment.

### BrdU staining

75,000 U87 cells were treated with BrdU (Thermo Fisher Scientific, Waltham, MA) for 2 h at 37 °C with 5% CO_2_, then cells were fixed in 4% paraformaldehyde for 15 min at room temperature. Cells were washed with PBS and permeabilized with 0.1% Triton-X 100 for 20 min. Cells were washed with 1 N HCL, 2 N HCL, and phosphate/citric acid buffer, then cells were stained with mouse BrdU (1:1000; Thermo Fisher Scientific, Waltham, MA) in PBS with 5% normal goat serum and 0.1% Triton-X 100 overnight at 4 °C. The next day, cells were washed in PBS and stained with goat anti-mouse Alexa Fluor 488 secondary antibody (1:500, Thermo Fisher Scientific, Waltham, MA) for 1 h at room temperature, then the cells were washed and stored in PBS. DAPI (Thermo Fisher Scientific, Waltham, MA) was added to the cells at a final concentration of 1 µM for 10 min, then the cells were visualized on a Nikon Ti Eclipse fluorescent microscope. The number of cells displaying BrdU staining was quantified using Image Pro Plus software, and the data was normalized for direct comparisons across experiments.

### Co-cultivation of U87 cells with human and mouse macrophages

100,000 U87 cells were plated in a 12 well plate, and cells were transfected 24 h later with Cas9-g82/165 + HDR or lipofectamine only. 8 h post transfection, 300,000 human SC macrophages or mouse bone marrow derived macrophages were added to U87 cells, and the co-cultures were allowed to incubate for 48 h to differentiate macrophages into tumor associated macrophages (TAMs).

### Enzyme-Linked Immunosorbent Assay (ELISA)

ELISA was performed on culture media from co-cultured U87 + BMM cells to determine the secretion of cytokines using ELISA kits purchased from Thermo Fisher Scientific (Waltham, MA). Cells were prepared and transfected as written in “Flow cytometry” section, and ELISAs were performed according to the manufacturer’s protocol. The data was collected on a FlexStation 3 Multi-Mode Microplate Reader (Molecular Devices, San Jose, CA) at 450 nm and 570 nm, and the background absorbance taken at 570 nm was subtracted from the absorbance read at 450 nm.

### Flow cytometry

For co-cultured cells, SC cells were removed from suspension and washed with PBS twice. U87 cells were then removed using Trypsin-EDTA (Thermo Fisher Scientific, Waltham, MA), washed twice in PBS, and combined with SC cells. For co-cultures containing BMM cells, U87 cells were removed using Trypsin-EDTA, followed by scraping the BMM cells with a cell scraper that were combined with the U87 cells. Co-cultured cells were washed in PBS twice after removal from the plate, blocked in 3% BSA + PBS for 20 min, then cells were stained with antibodies as listed in Table 2 (Thermo Fisher Scientific, Waltham, MA) for 30 min. Cells were washed twice in 1% BSA + PBS, and then fixed in 2% paraformaldehyde (Sigma-Aldrich, St Louis, MO) in PBS. Data on 5 × 10^5^ cells were collected on a Gallios Flow Cytometer (Beckman Coulter, Brea, CA), and the data was analyzed using FlowJo software (FlowJo, LLC, Ashland, OR).

**Table 2:** Antibodies for flow cytometry.

Antigen	Conjugate	Clone
Arginase I	Alexa Fluor 488	A1exF5
CD11b	Alexa Fluor 700	M1/70
CD204	FITC	C068C2
CD206	FITC	C068C2
CD45	Pacific Orange	30-F11
CD80	APC	16-10A1
F4/80	APC	BM8
Ly6C	PerCP-Cy 5.5	HK1.4
Ly6G	Alexa Fluor 700	1A8-Ly6g
PD-L1	PE	MIH1
PD-L1	PE	MIH5

### Cas9-g70/166 + HDR knockout mouse PD-L1 in vivo

NPs developed in our lab^46,47^ were used to deliver Cas9-g70/166 + HDR for in vivo studies. NPs were loaded with 3ug Cas9-g70/166 plasmid DNA with the HDR template at an N/P ratio of 3.1, and allowed to incubate for 30 min at room temperature. Cas9-g70/166 loaded NPs (0.1 mL) were injected in Balb/c mice via i.v injection at a final concentration of 1.5ug Cas9-g70/166 and 12.5 µM HDR template per mL of mouse blood (approximately 2mLs). Mice received a total of 3 doses at days 1, 3, and 6, then mice were sacrificed on day 9. Mice injected with unloaded nanoparticles served as the control.

All animal experiments were carried out in strict accordance with the recommendations in the Guide for the Care and Use of Laboratory Animals of the National Research Council. The study was reported in accordance with ARRIVE guidelines and approved by the Institutional Animal Care and Use Committee. All mice were at 4–8 weeks and were raised and maintained by the Laboratory Animal Resource Center at TTUHSC—El Paso.

### Statistical analysis

Statistical analysis including a t-test or a one-way ANOVA followed by Tukey’s post hoc analysis was carried out in SPSS (IBM, Armonk, NY). Variance between groups was similar and the data was normally distributed. All error bars are expressed as the standard error of the mean (SEM). All experiments were performed in triplicate, and the data was normalized for direct comparisons.

## Supplementary Information

Supplementary Table 1.

Supplementary Table 2.

Supplementary Table 3.

Supplementary Table 4.

Supplementary Figure 1.

Supplementary Figure 2.

Supplementary Figure 3.

Supplementary Figure 4.

Supplementary Figure 5.

Supplementary Figure 6.

Supplementary Figure 7.

Supplementary Figure 8.

Supplementary Figure 9.

Supplementary Legends.
