# Supplementary material for: Dual-sgRNA CRISPR/Cas9 knockout of PD-L1 in human U87 glioblastoma tumor cells inhibits proliferation, invasion, and tumor-associated macrophage polarization
Source: Sci Rep. 2022 Feb 14;12:2417. doi: 10.1038/s41598-022-06430-1 (PMC8844083; doi:10.1038/s41598-022-06430-1)
Supplement: Supplementary file 1 — Supplementary Table 1. [file 41598_2022_6430_MOESM1_ESM.docx]

**Supplemental Table 1: Off-target analysis for human g82**

|  | **Sequence** | **PAM** | **Score** | **#MM** | **Gene** | **Locus** |
| --- | --- | --- | --- | --- | --- | --- |
|  | *GGTTCCCAAGGACCTATATG* | *TGG* |  |  | *PD-L1* |  |
| 1 | GATTGCCAAGGACCTATATA | GAG | 11 | 3 |  | chr2:+196802606 |
| 2 | GGTTCCC-AGGACCTATATA | AGG | 16 | 2 |  | chr11:+115433647 |
| 3 | GGTAGCCAAGAAACTATATG | CAG | 24 | 4 |  | chrX:-42870751 |
| 4 | GGTTACCAAGGACCTAGATG | TAG | 32 | 2 |  | chr6:-91636265 |
| 5 | TGTGCCCAAGGACCTAAATG | AGG | 33 | 3 |  | chr14:+89205388 |
| 6 | CTTTCCCAAGAACCTAGATG | TAG | 34 | 4 |  | chr5:-69531007 |
| 7 | TGTTCCCCAGGACCTAGATG | GGG | 36 | 3 |  | chr2:+20103981 |
| 8 | GGTTGCCAATGCCCTATATA | TGG | 37 | 4 |  | chr8:+135385447 |
| 9 | TGTGCCCAGGGACCTAGATG | AGG | 39 | 4 |  | chr12:-80513592 |
| 10 | TGTTTCCAAGATCCTATATG | GGG | 39 | 4 |  | chr13:-30933161 |
| 11 | TGTGCCCAGGGACCTACATG | AGG | 46 | 4 |  | chr4:-32473710 |
| 12 | GTTTCCCCAGGACCTAGATA | AGG | 47 | 4 |  | chrX:-132452487 |
| 13 | GGTTCACTGAGACCTATATG | AAG | 49 | 4 |  | chr8:+88221838 |
| 14 | TGTGCCCAAGGACCTATGTA | AGG | 49 | 4 |  | chr13:-81676457 |
| 15 | AGTTTCCAAAGACCTAAATG | GGG | 52 | 4 |  | chr8:+38504751 |
| 16 | TGTGCCCACGGACCTAGATG | AGG | 52 | 4 |  | chr11:-86413394 |
| 17 | TGTGCCCACGGACCTAGATG | AGG | 52 | 4 |  | chr18:+68152409 |
| 18 | GACTCCCAAGGCCCTAAATG | GAG | 52 | 4 |  | chr14:-31965588 |
| 19 | GTTTTCCAAGAACCTTTATG | TGG | 52 | 4 |  | chr7:-66889739 |
| 20 | TGTGCCCACGGACCTAAATG | AGG | 53 | 4 |  | chr13:+84720127 |
