# Supplementary material for: Dual-sgRNA CRISPR/Cas9 knockout of PD-L1 in human U87 glioblastoma tumor cells inhibits proliferation, invasion, and tumor-associated macrophage polarization
Source: Sci Rep. 2022 Feb 14;12:2417. doi: 10.1038/s41598-022-06430-1 (PMC8844083; doi:10.1038/s41598-022-06430-1)
Supplement: Supplementary file 2 — Supplementary Table 2. [file 41598_2022_6430_MOESM2_ESM.docx]

**Supplemental Table 2: Off-target analysis for human g165**

|  | **Sequence** | **PAM** | **Score** | **#MM** | **Gene** | **Locus** |
| --- | --- | --- | --- | --- | --- | --- |
|  | *TGGCTGCACTAATTGTCTAT* | *TGG* |  |  | *PD-L1* |  |
| 1 | TAGCTGC-CTAGTTGTCTAT | GGG | 22 | 3 |  | chr2:+137216882 |
| 2 | TTACTGAACTAAATGTCTAT | AGG | 27 | 4 |  | chr3:+116243739 |
| 3 | TGGCTGAACTAATTGTTTAT | GAG | 30 | 2 |  | chr8:+13852512 |
| 4 | TGGCGGCAC-AATTATCTAT | TAG | 30 | 3 |  | chr5:-100641657 |
| 5 | TGGGTGCACTAATTATCTTT | TAG | 31 | 3 |  | chr2:+53763010 |
| 6 | TGGCTGTACTGATTGTCTAT | AGG | 32 | 2 |  | chrX:-71904237 |
| 7 | TTGTTGCAGTAATTATCTAT | AAG | 37 | 4 |  | chr15:+79586628 |
| 8 | TAGCTGCTCTCATTGTCTAT | TAG | 38 | 3 |  | chr19:+39046187 |
| 9 | TAGCTGCTCTCATTGTCTAT | TAG | 38 | 3 |  | chr4:-70520210 |
| 10 | GGGATGCA-TAATTGTCTAT | AGG | 38 | 3 |  | chr3:+118647200 |
| 11 | TAGCTGC-CTCATTGTCTAT | AAG | 40 | 3 |  | chr17:+63890380 |
| 12 | TAGCTGC-CTCATTGTCTAT | AGG | 40 | 3 |  | chr17:+63867511 |
| 13 | AGGCTATACTGATTGTCTAT | TAG | 43 | 4 |  | chr13:+30035581 |
| 14 | TGGCCCCCCTAATTGACTAT | AAG | 45 | 4 |  | chrX:+42101157 |
| 15 | TGGCTGCA-TAAATGTCTAT | AAG | 46 | 2 |  | chr3:+183610768 |
| 16 | TGG-AGCACTAATTGTCAAT | TGG | 46 | 3 |  | chr14:+90251795 |
| 17 | TGGCTGCACTAAATGTCTAA | AAG | 48 | 2 |  | chr2:+167326032 |
| 18 | TGGGTAAACTAATTGTTTAT | TGG | 49 | 4 |  | chr3:-26549815 |
| 19 | TCTCTGAACTAAATGTCTAT | TAG | 49 | 4 |  | chr3:-108562531 |
| 20 | TTGCTGACCTAATTGTCAAT | TAG | 49 | 4 |  | chr15:+61957662 |
