# Supplementary material for: Dual-sgRNA CRISPR/Cas9 knockout of PD-L1 in human U87 glioblastoma tumor cells inhibits proliferation, invasion, and tumor-associated macrophage polarization
Source: Sci Rep. 2022 Feb 14;12:2417. doi: 10.1038/s41598-022-06430-1 (PMC8844083; doi:10.1038/s41598-022-06430-1)
Supplement: Supplementary file 3 — Supplementary Table 3. [file 41598_2022_6430_MOESM3_ESM.docx]

**Supplemental Table 3: Off-target analysis for mouse g70**

|  | **Sequence** | **PAM** | **Score** | **#MM** | **Gene** | **Locus** |
| --- | --- | --- | --- | --- | --- | --- |
|  | *GTTTACTATCACGGCTCCAA* | *AGG* |  |  | *PD-L1* |  |
| 1 | GCTTACAATGCACGGCTCCAA | AAG | 30 | 3 |  | chr15:-46278195 |
| 2 | TTTTGCTCTCA-GGCTCCAA | CAG | 31 | 4 |  | chr20:+39121039 |
| 3 | G-TTGCTTTCATGGCTCCAA | GAG | 32 | 4 |  | chr13:+49840374 |
| 4 | CTGTACAACTCACGGCTCCAA | AGG | 36 | 4 |  | chr15:+51524957 |
| 5 | GCTTACATATCACAGCTCCAA | AGG | 37 | 3 |  | chr10:+76327437 |
| 6 | CTTT-CTGTCCCGGCTCCAA | TAG | 38 | 4 |  | chr7:-149784568 |
| 7 | GTTTACT-TCACAGCTCCTA | AAG | 39 | 3 |  | chr11:-77027210 |
| 8 | GTTTACAACCA-GGCTCCAA | GAG | 42 | 3 |  | chr6:-79428776 |
| 9 | GTTTTCT-TCATGGCTCCTA | TAG | 42 | 4 |  | chr14:-78311793 |
| 10 | GTTT-CTCTCATGGCTCCTA | AGG | 43 | 4 |  | chr1:-230000557 |
| 11 | ATTTCCTTTCA-GGCTCCAA | AGG | 45 | 4 |  | chr4:+21838157 |
| 12 | GTGTACTGT-ACAGCTCCAA | GAG | 47 | 4 |  | chr11:+30421807 |
| 13 | CTTTTCTACCACGGCTCCAG | TAG | 48 | 4 |  | chr13:-107297055 |
| 14 | GCTTAC-ACCACAGCTCCAA | AGG | 48 | 4 |  | chr1:+234933116 |
| 15 | GTTCAATTTCA-GGCTCCAA | CAG | 49 | 4 |  | chr7:-42458617 |
| 16 | GTTAACTATC-TGGCTCCAA | TAG | 50 | 3 |  | chr1:+211035811 |
| 17 | CTTTAATCTCAAGGCTCCAA | TGG | 52 | 4 |  | chr5:-43116579 |
| 18 | GTTTA--ACCACGGCTCCAA | AGG | 53 | 3 |  | chr9:-23011890 |
| 19 | ATTT-CTTTCACGTCTCCAA | GGG | 55 | 4 |  | chrX:+134512673 |
| 20 | TCTTAC-ATCACGGCTGCAA | GAG | 57 | 4 |  | chr4:+62613968 |
