# Supplementary material for: Dual-sgRNA CRISPR/Cas9 knockout of PD-L1 in human U87 glioblastoma tumor cells inhibits proliferation, invasion, and tumor-associated macrophage polarization
Source: Sci Rep. 2022 Feb 14;12:2417. doi: 10.1038/s41598-022-06430-1 (PMC8844083; doi:10.1038/s41598-022-06430-1)
Supplement: Supplementary file 4 — Supplementary Table 4. [file 41598_2022_6430_MOESM4_ESM.docx]

**Supplemental Table 4: Off-target analysis for mouse g166**

|  | **Sequence** | **PAM** | **Score** | **#MM** | **Gene** | **Locus** |
| --- | --- | --- | --- | --- | --- | --- |
|  | *AGTACACCACTAACGCAAGC* | *AGG* |  |  | *PD-L1* |  |
| 1 | AGCACAA-ACTAACGCAAGC | TAG | 8 | 3 |  | chr5:-67235631 |
| 2 | AGCA-ACTACTAACACAAGC | AGG | 13 | 4 |  | chr17:-46536176 |
| 3 | AGCA-ACTACTAACACAAGC | AGG | 13 | 4 |  | chr17:-46318311 |
| 4 | AGCA-ACTACTAACACAAGC | AGG | 13 | 4 |  | chr17:+64873113 |
| 5 | ATTATACCACTAACATAAGC | TAG | 33 | 4 |  | chr15:-34599060 |
| 6 | AGTT-ACCCTTAACGCAAGC | AAG | 36 | 4 |  | chr2:-100418018 |
| 7 | CGTA-ACCACTAACAAAAGC | AAG | 38 | 4 |  | chr8:+102037113 |
| 8 | TTTACAGCCCCTAACGCAAGC | AGG | 44 | 4 |  | chr3:-187288972 |
| 9 | AGTCC-CCATTAACACAAGC | AGG | 45 | 4 |  | chr10:-45597259 |
| 10 | TGTA---CACTAACGCAAGC | CAG | 45 | 4 |  | chrX:+110833953 |
| 11 | AGTG-ACCACTAATGCAAGC | TGG | 47 | 3 |  | chr4:-53902163 |
| 12 | TGGACAACACTAAC-CAAGC | AAG | 47 | 4 |  | chr8:+86283715 |
| 13 | AGCACAGCACTAACGAAAGC | CAG | 48 | 3 |  | chr7:+140380014 |
| 14 | AGTAAA--TCTAACGCAAGC | TGG | 48 | 4 |  | chr11:+105721948 |
| 15 | AGCAGACCACAAAC-CAAGC | CGG | 48 | 4 | LOC102724965 | chr2:+45650442 |
| 16 | AGT-CACCACTGACGCAACC | CAG | 49 | 3 |  | chr10:+29387754 |
| 17 | AGGAAAACACTAACGCAAGT | CAG | 49 | 4 |  | chr10:-64255805 |
| 18 | AGCA-ACCACAAACGCAGGC | CAG | 49 | 4 |  | chr14:+31355115 |
| 19 | AATAAACCACTAACA-AAGC | TGG | 50 | 4 |  | chr7:-118930974 |
| 20 | AGCAAACCACTAACGTAATC | TAG | 50 | 4 |  | chr2:+80078593 |
