# Supplementary figures and images for: Dual-sgRNA CRISPR/Cas9 knockout of PD-L1 in human U87 glioblastoma tumor cells inhibits proliferation, invasion, and tumor-associated macrophage polarization
Source: Sci Rep. 2022 Feb 14;12:2417. doi: 10.1038/s41598-022-06430-1 (PMC8844083; doi:10.1038/s41598-022-06430-1)

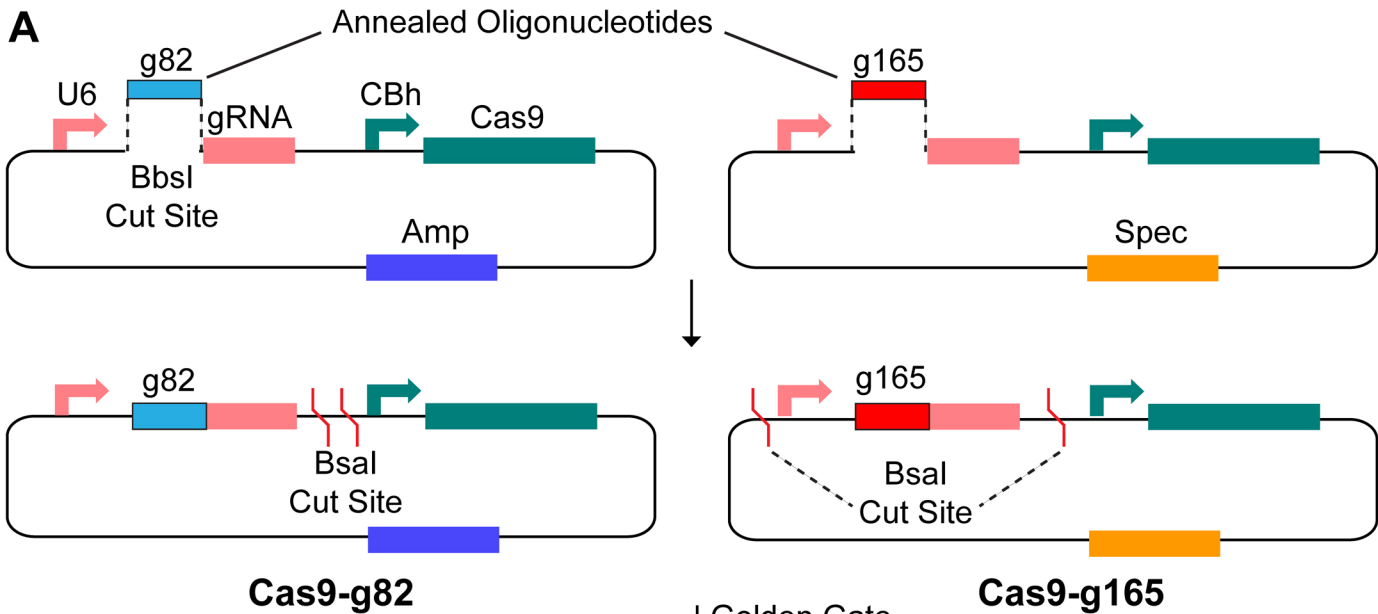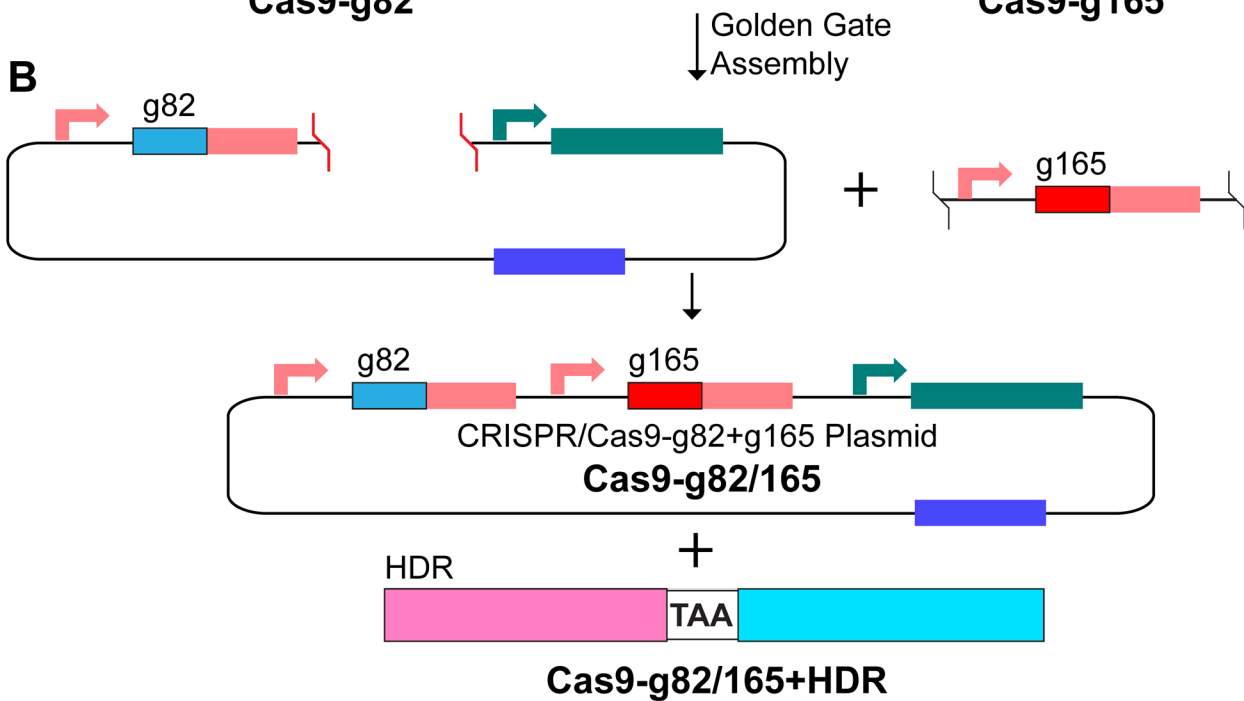

Supplement: Supplementary file 5 — Supplementary Figure 1. [file 41598_2022_6430_MOESM5_ESM.pdf]

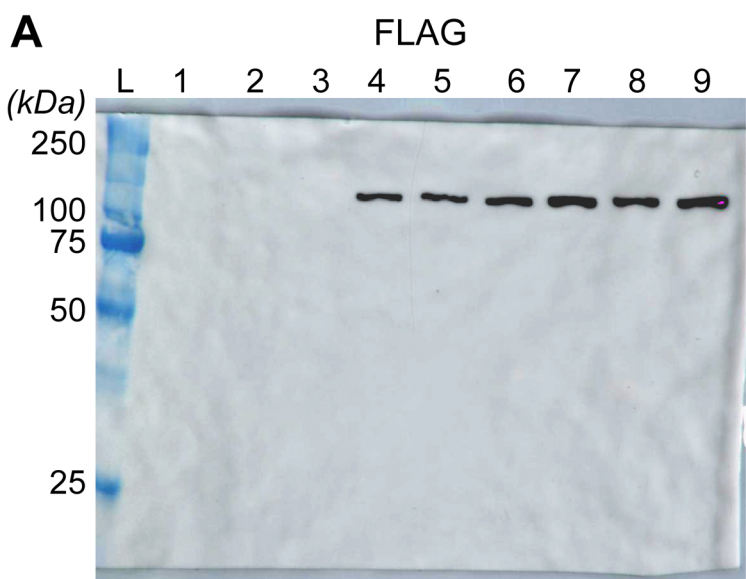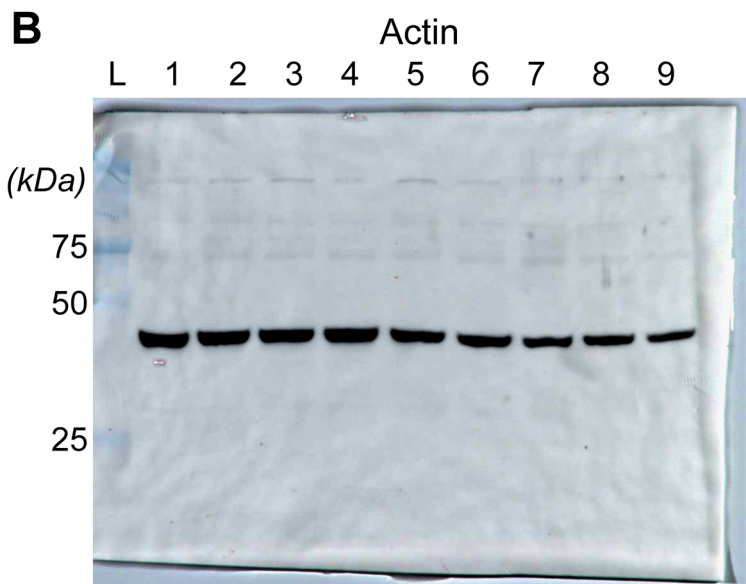

Supplement: Supplementary file 6 — Supplementary Figure 2. [file 41598_2022_6430_MOESM6_ESM.pdf]

**A**

PD-L1

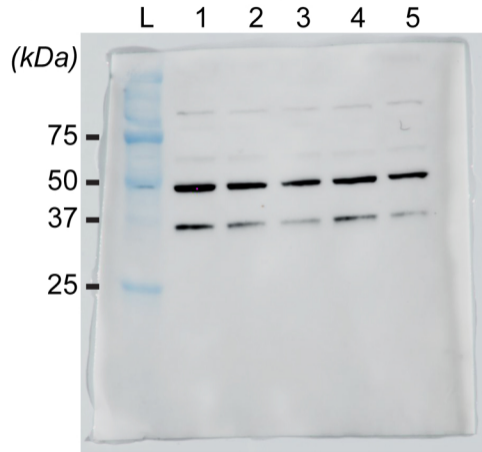**B**

Actin

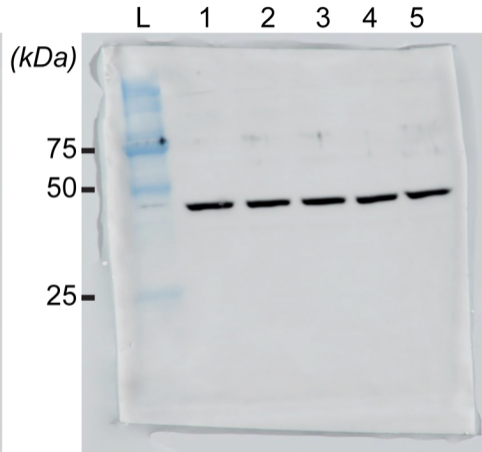**C**

FLAG

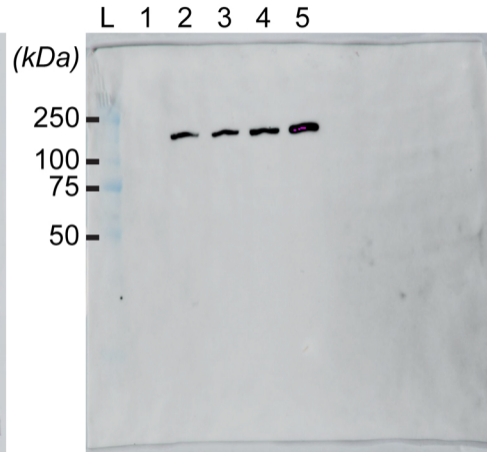

Supplement: Supplementary file 7 — Supplementary Figure 3. [file 41598_2022_6430_MOESM7_ESM.pdf]

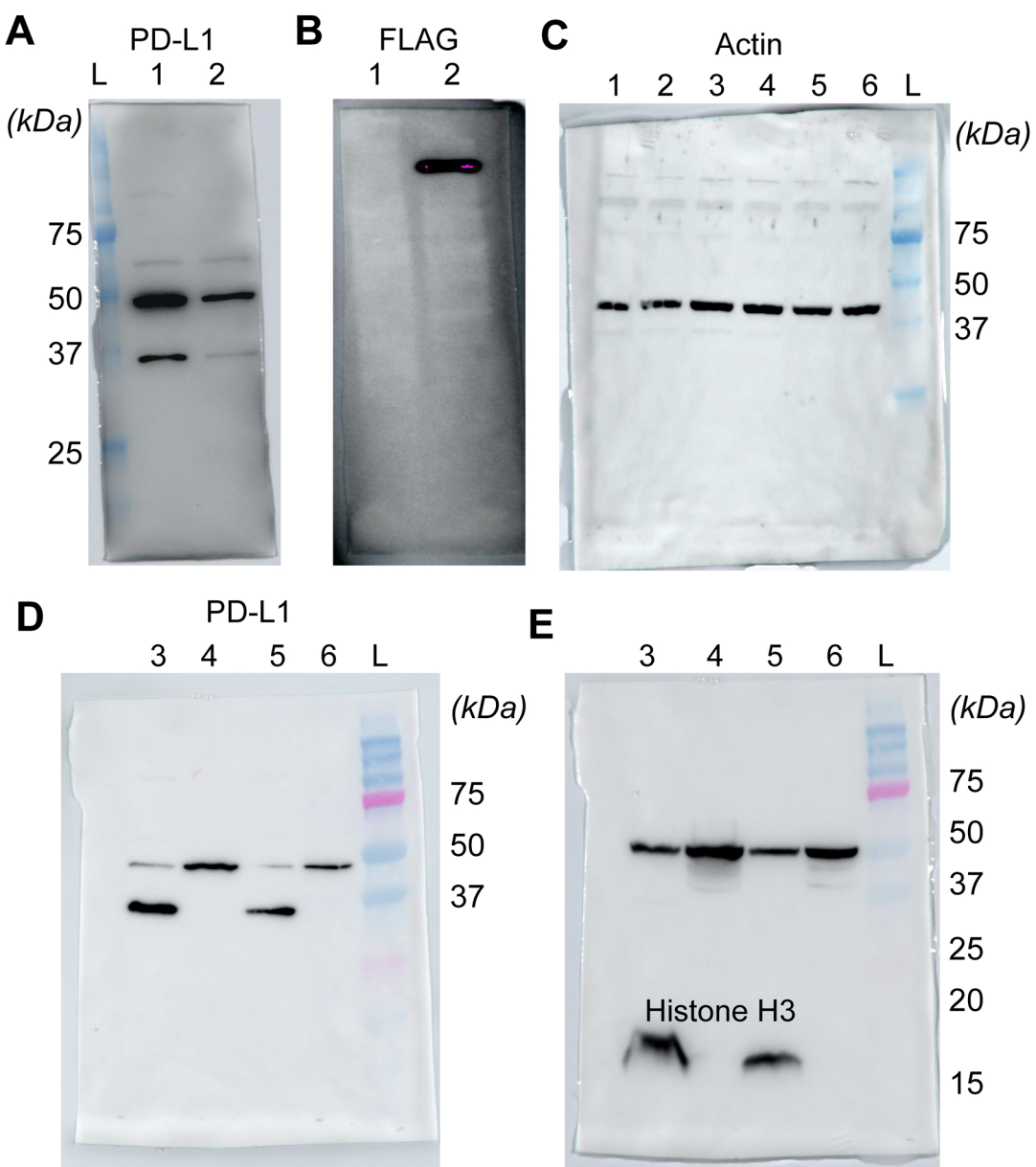

Supplement: Supplementary file 8 — Supplementary Figure 4. [file 41598_2022_6430_MOESM8_ESM.pdf]

Ctl

g82/165 + HDR

0 days

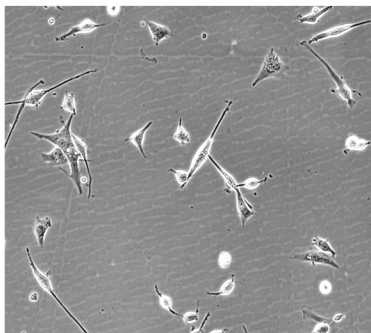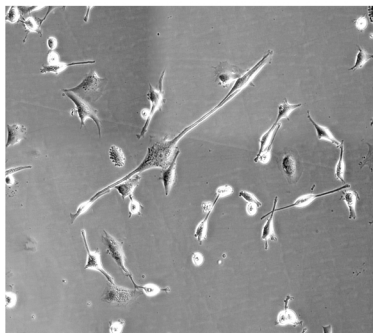

1

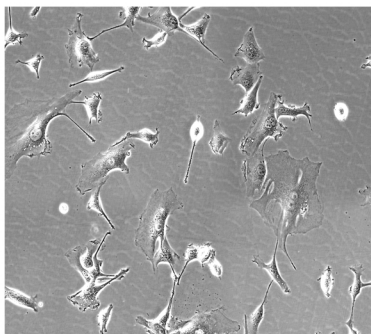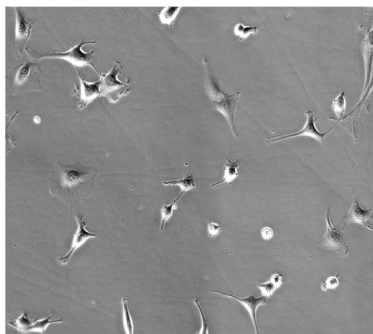

2

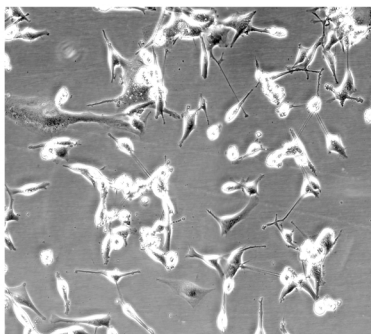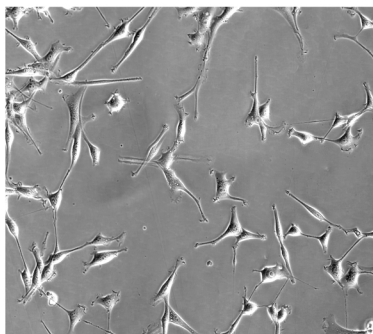

3

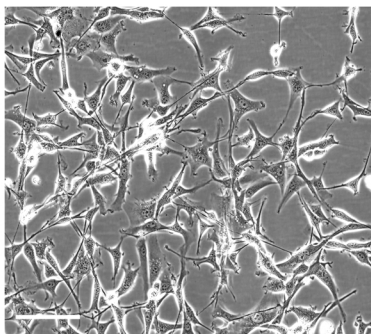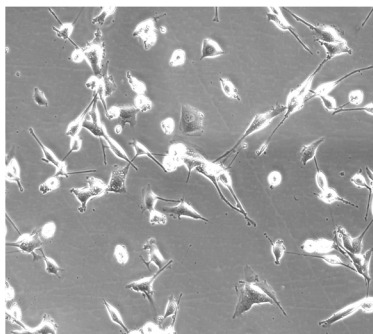

Supplement: Supplementary file 9 — Supplementary Figure 5. [file 41598_2022_6430_MOESM9_ESM.pdf]

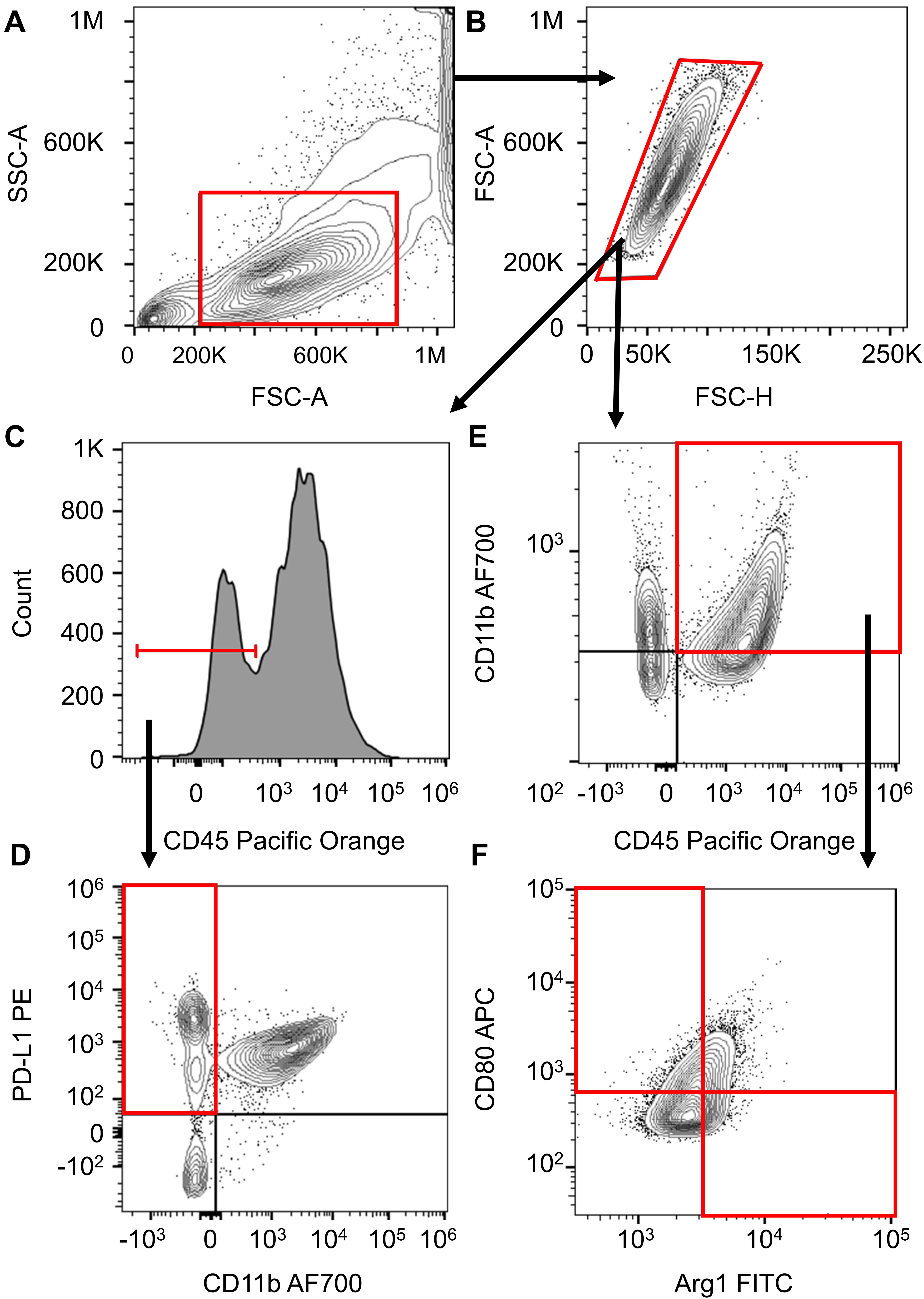

Supplement: Supplementary file 10 — Supplementary Figure 6. [file 41598_2022_6430_MOESM10_ESM.pdf]

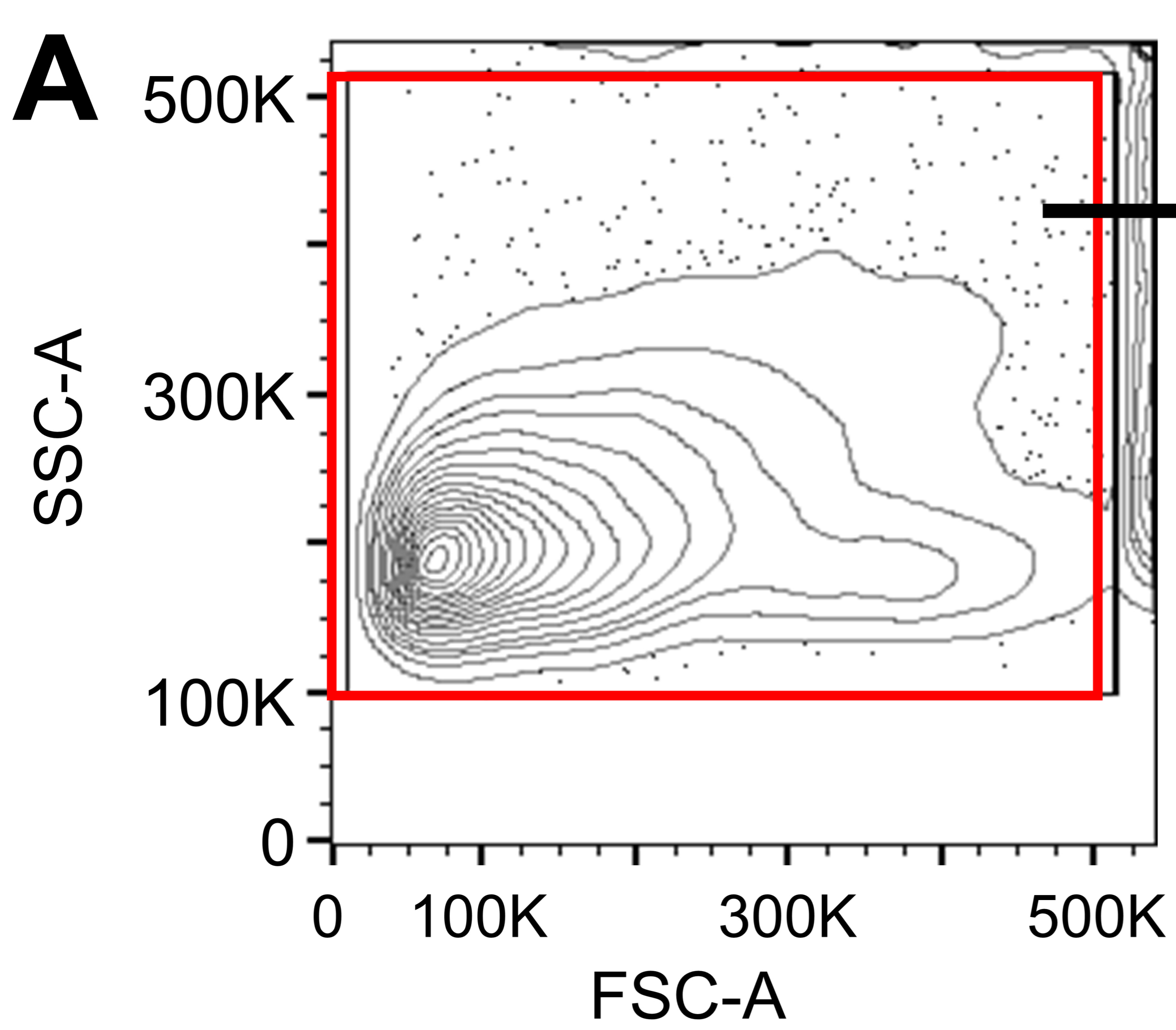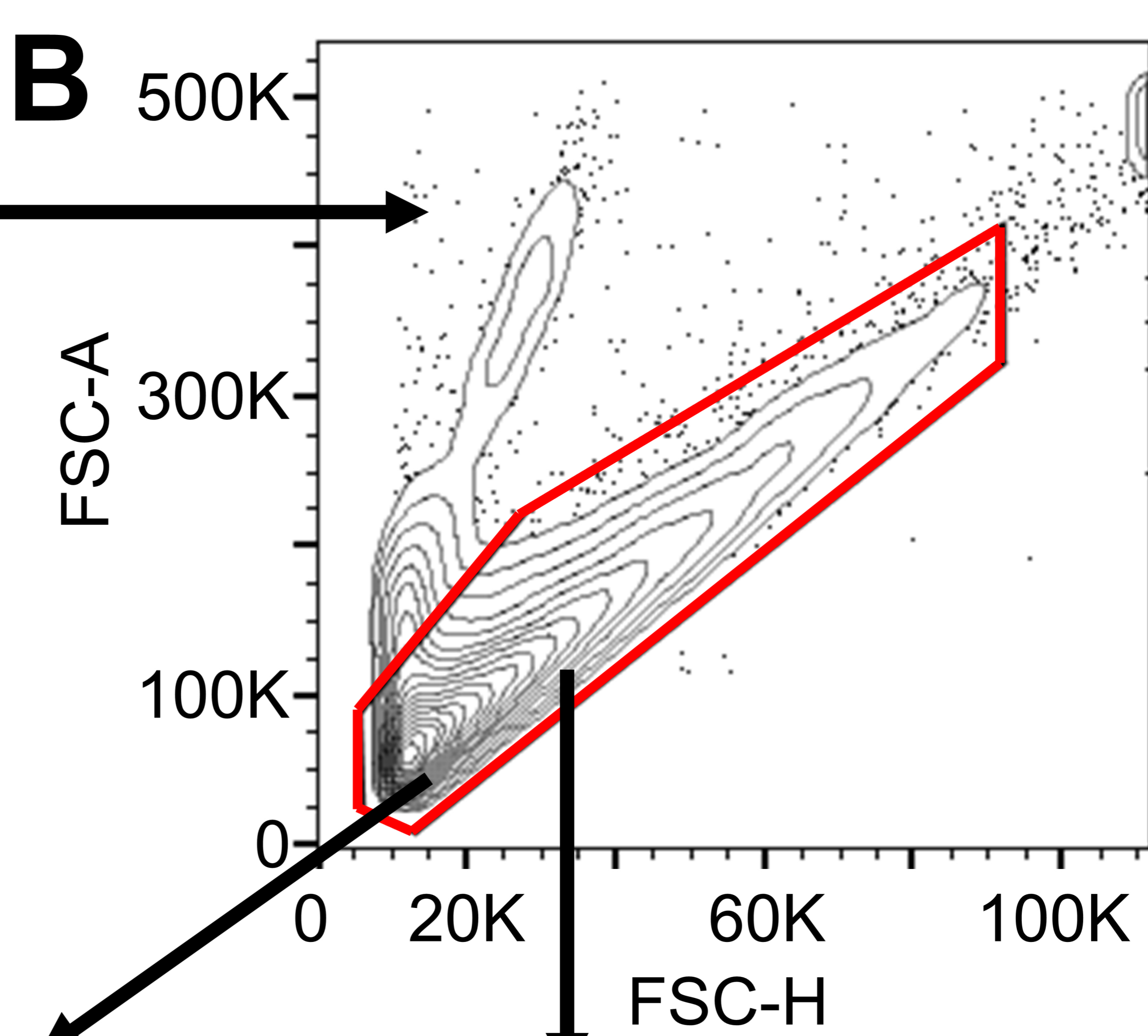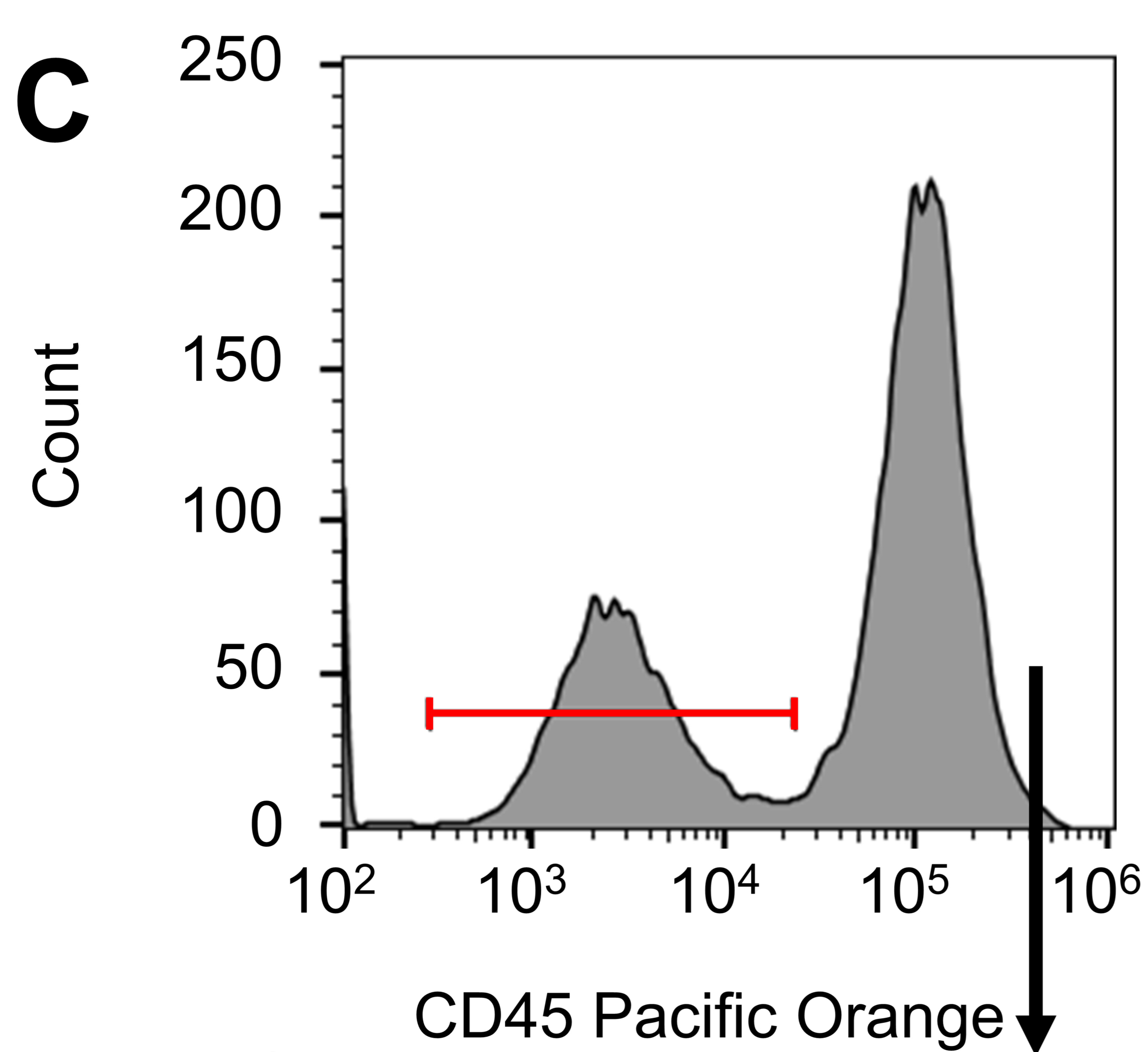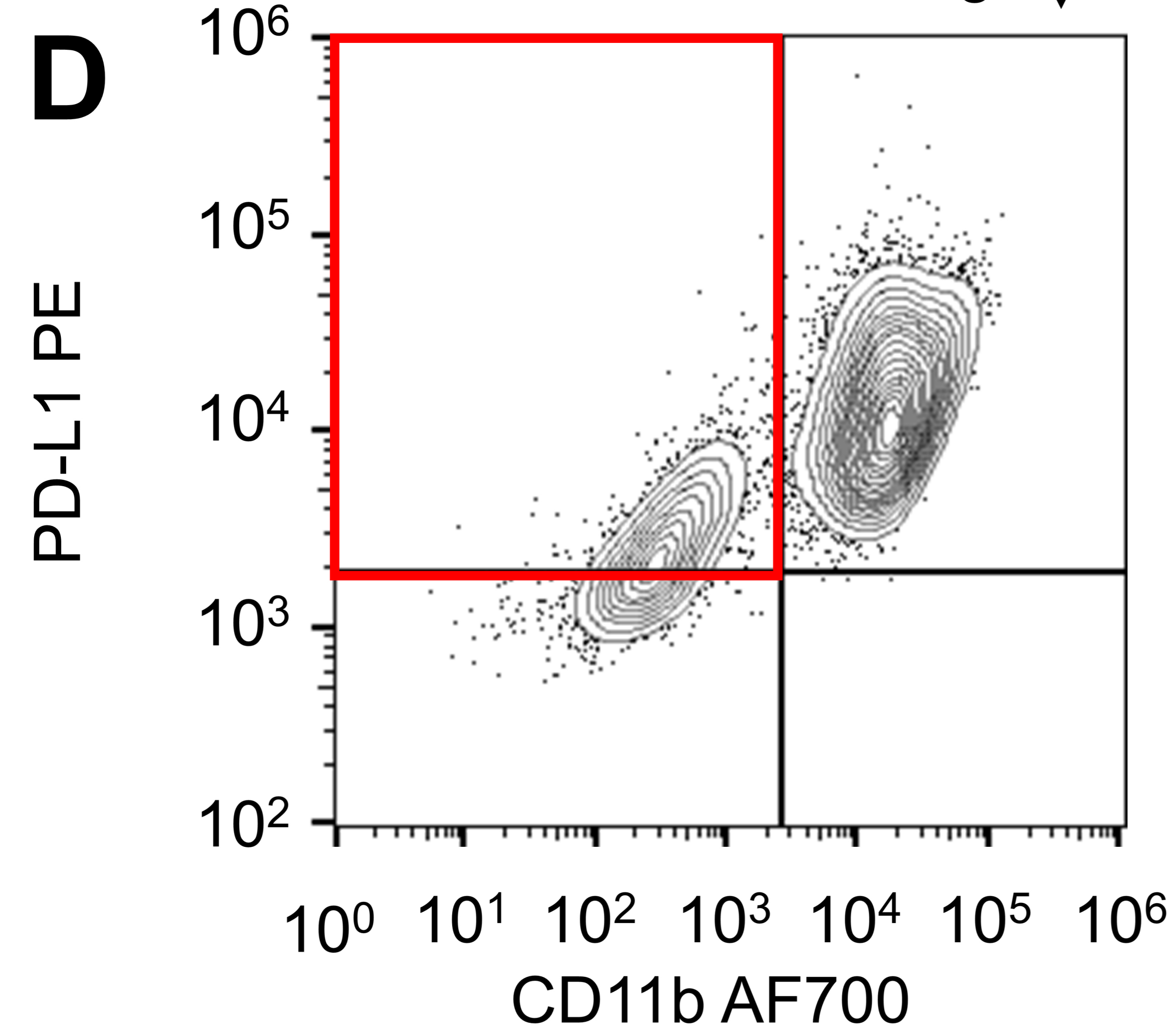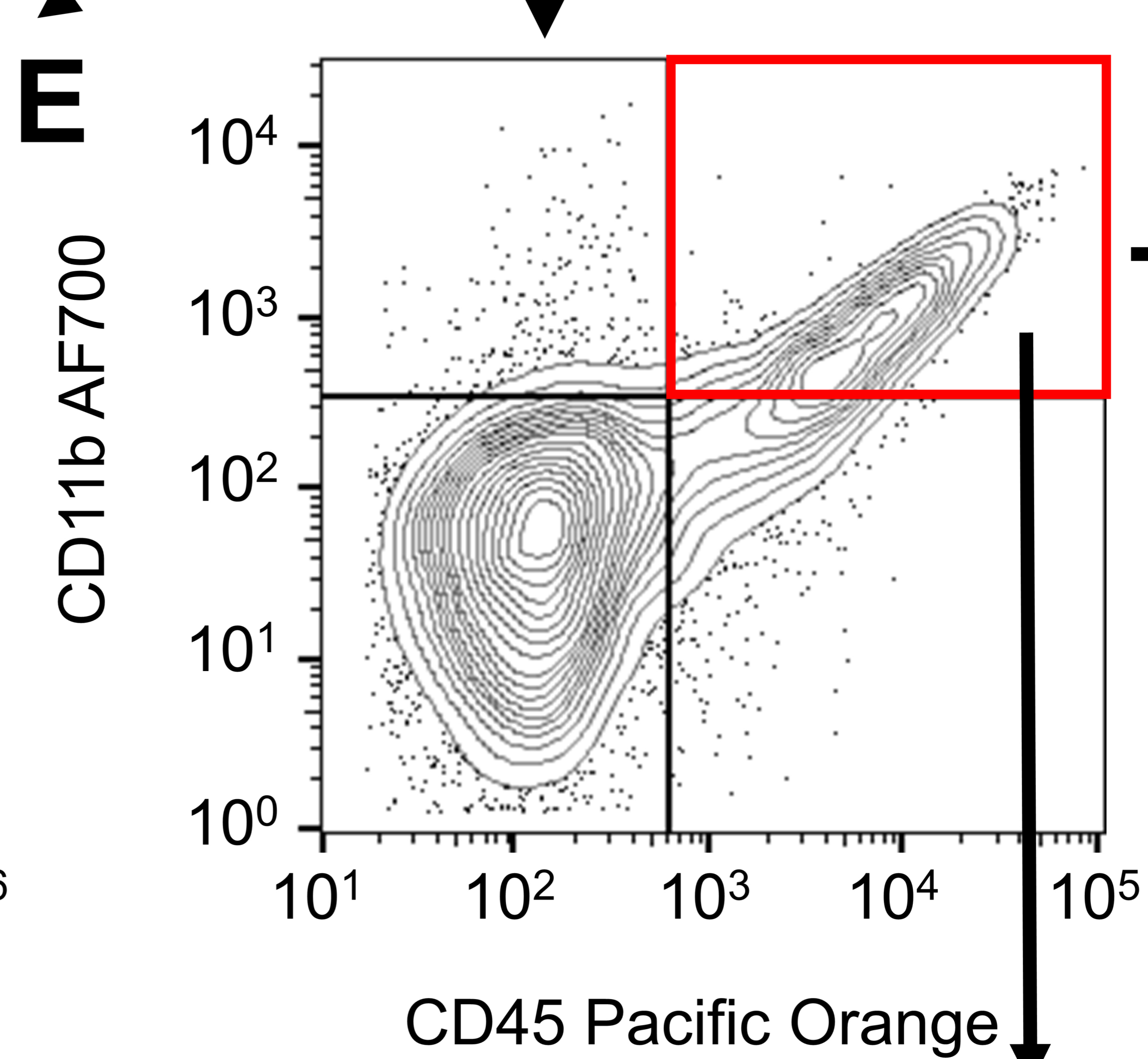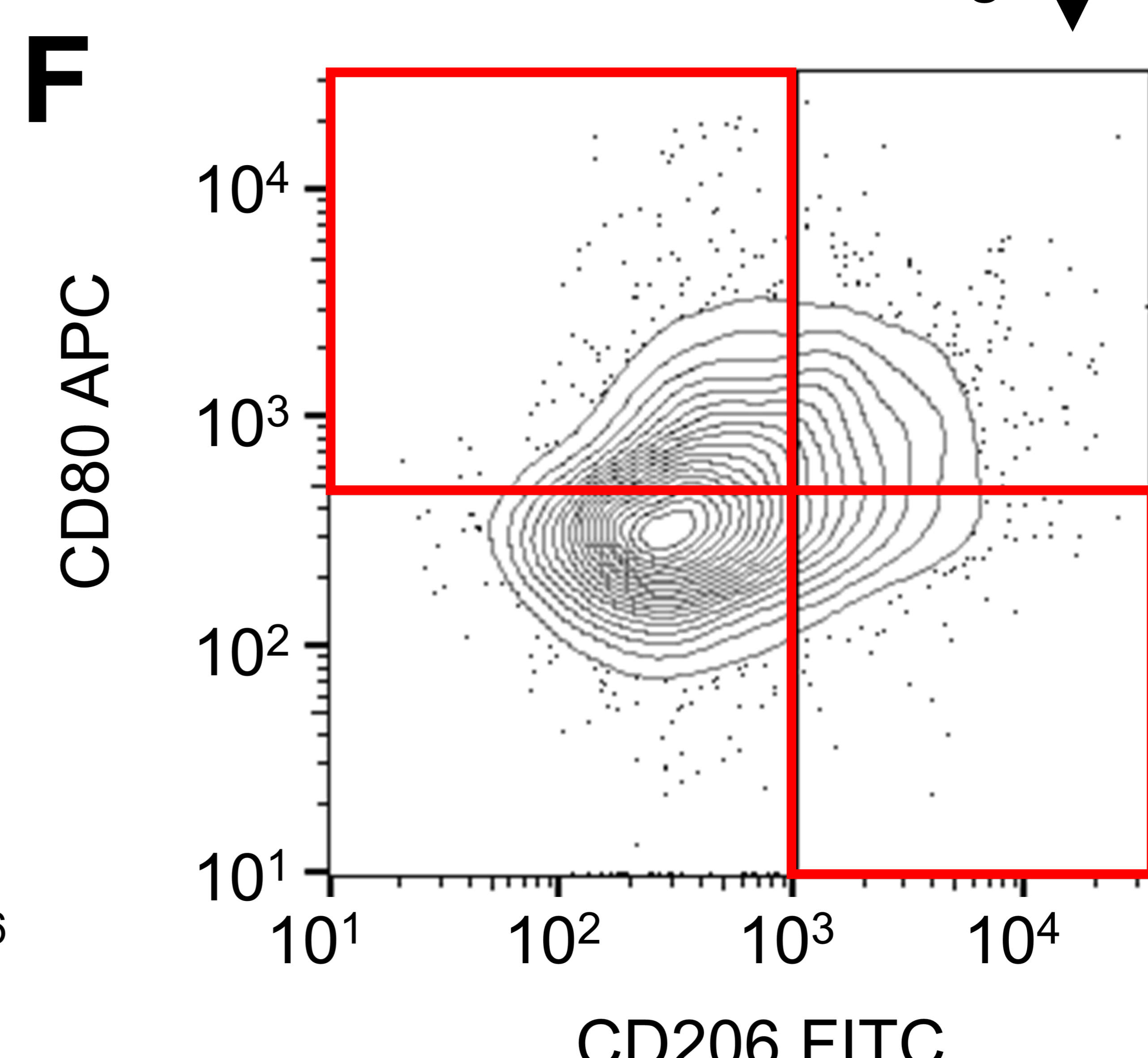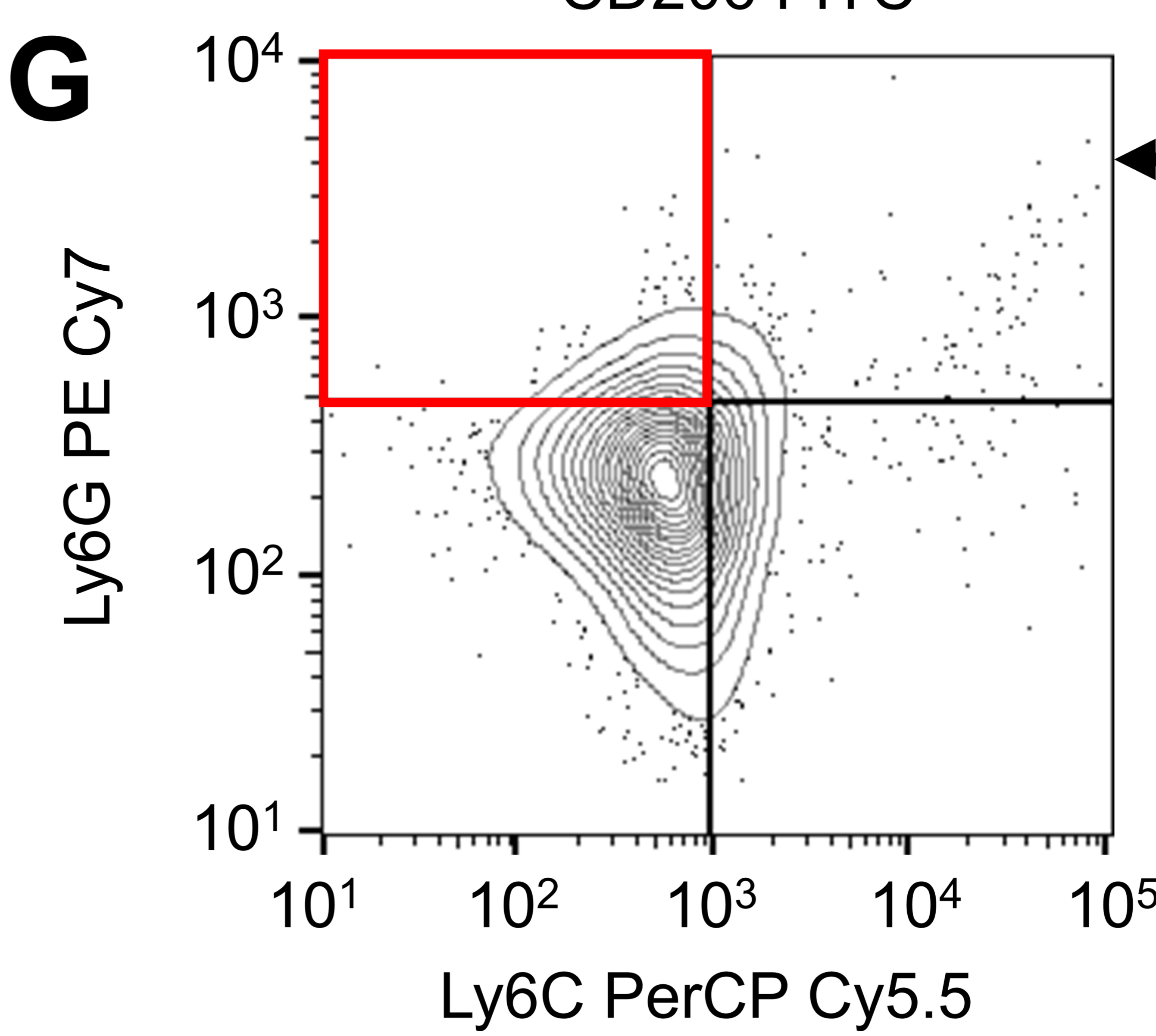

Supplement: Supplementary file 11 — Supplementary Figure 7. [file 41598_2022_6430_MOESM11_ESM.pdf]

**A**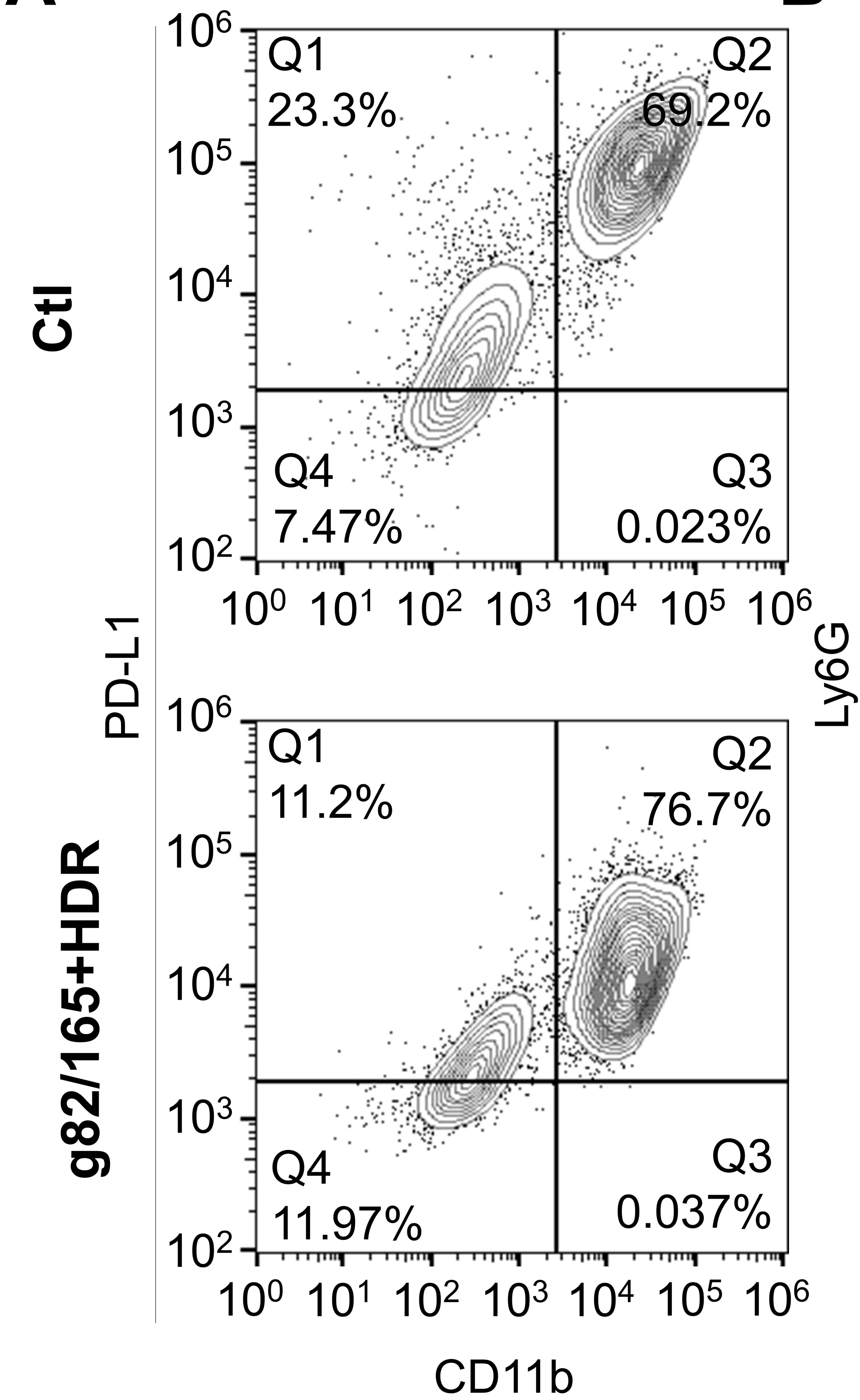**B**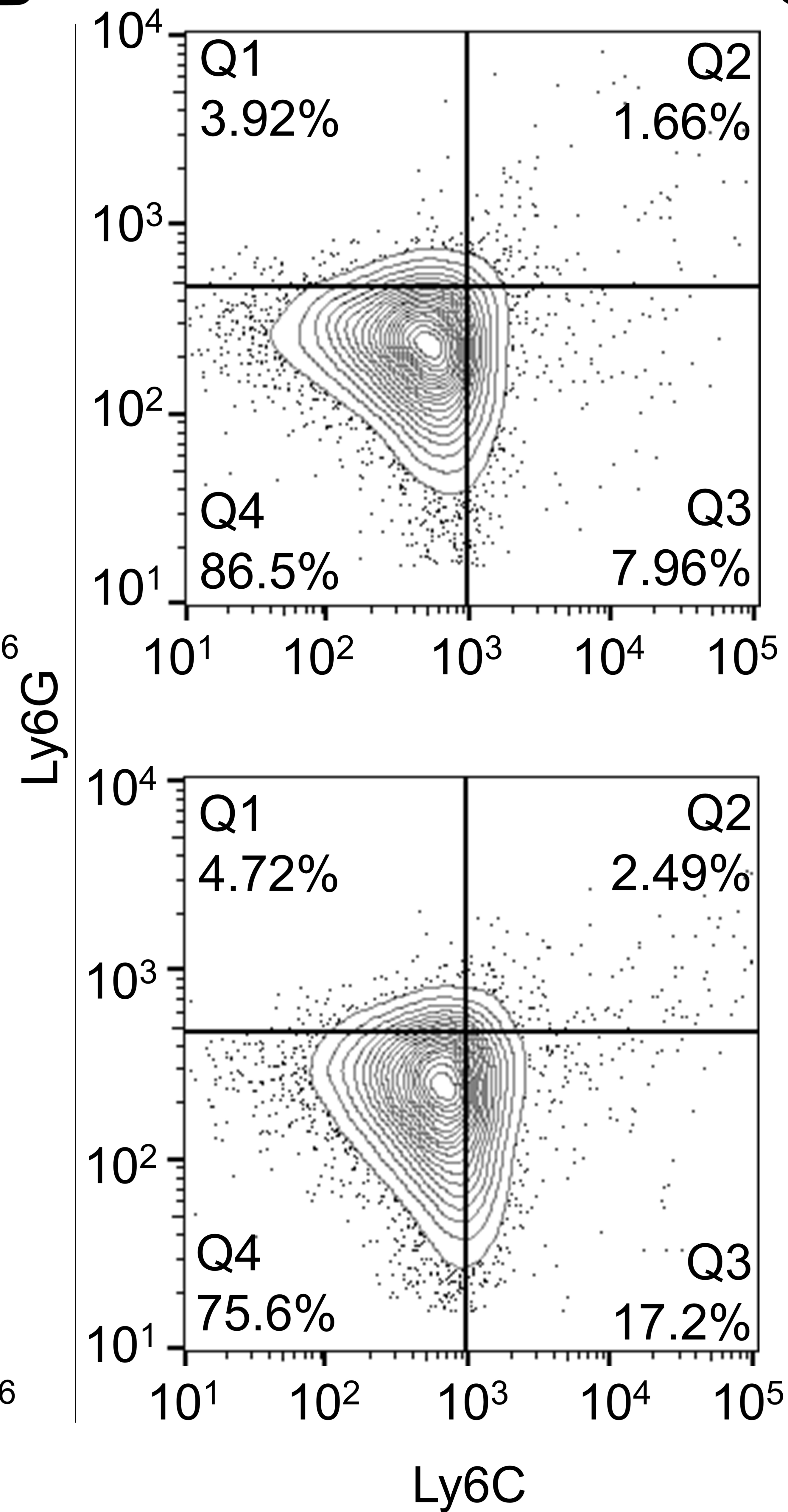**C**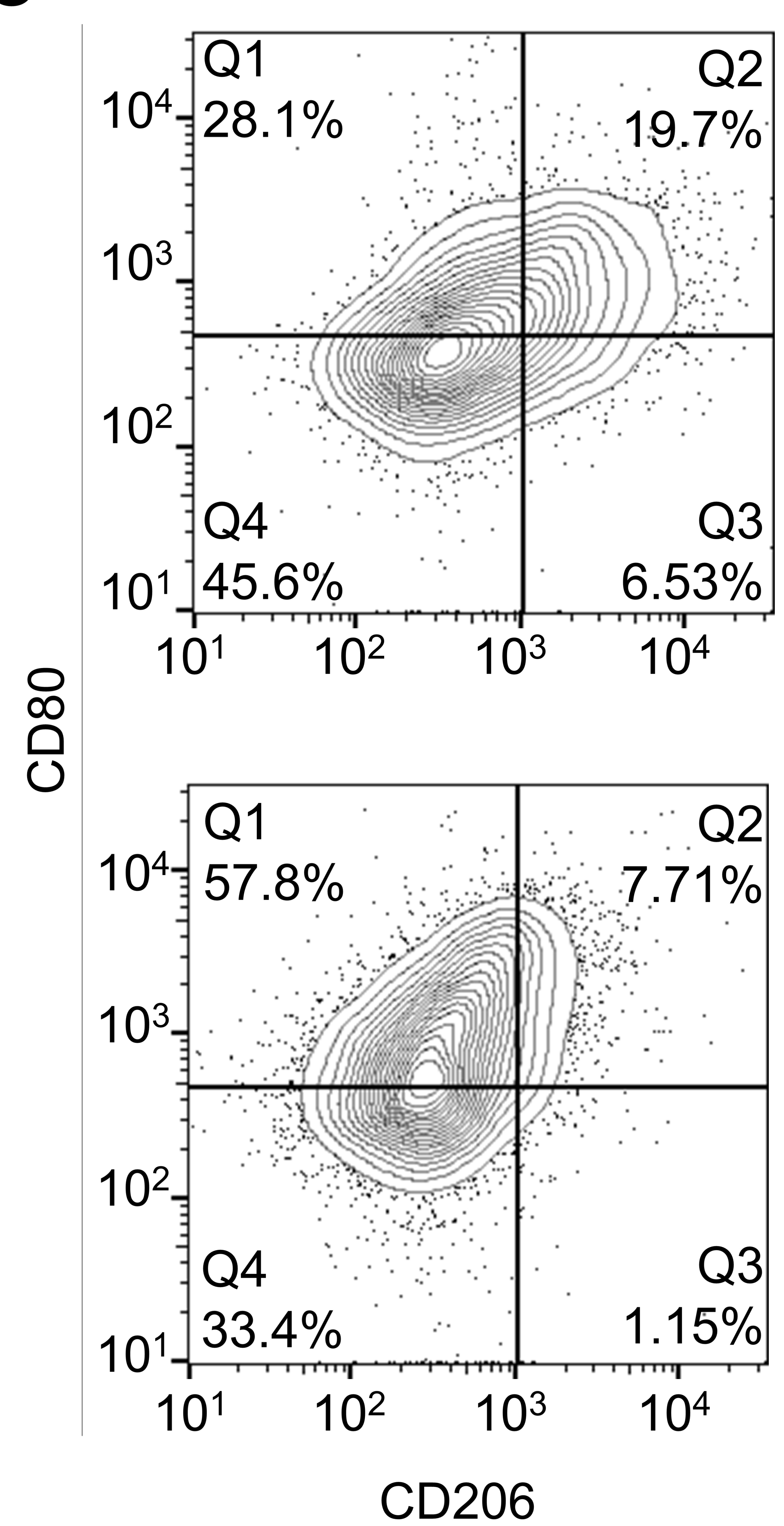**D**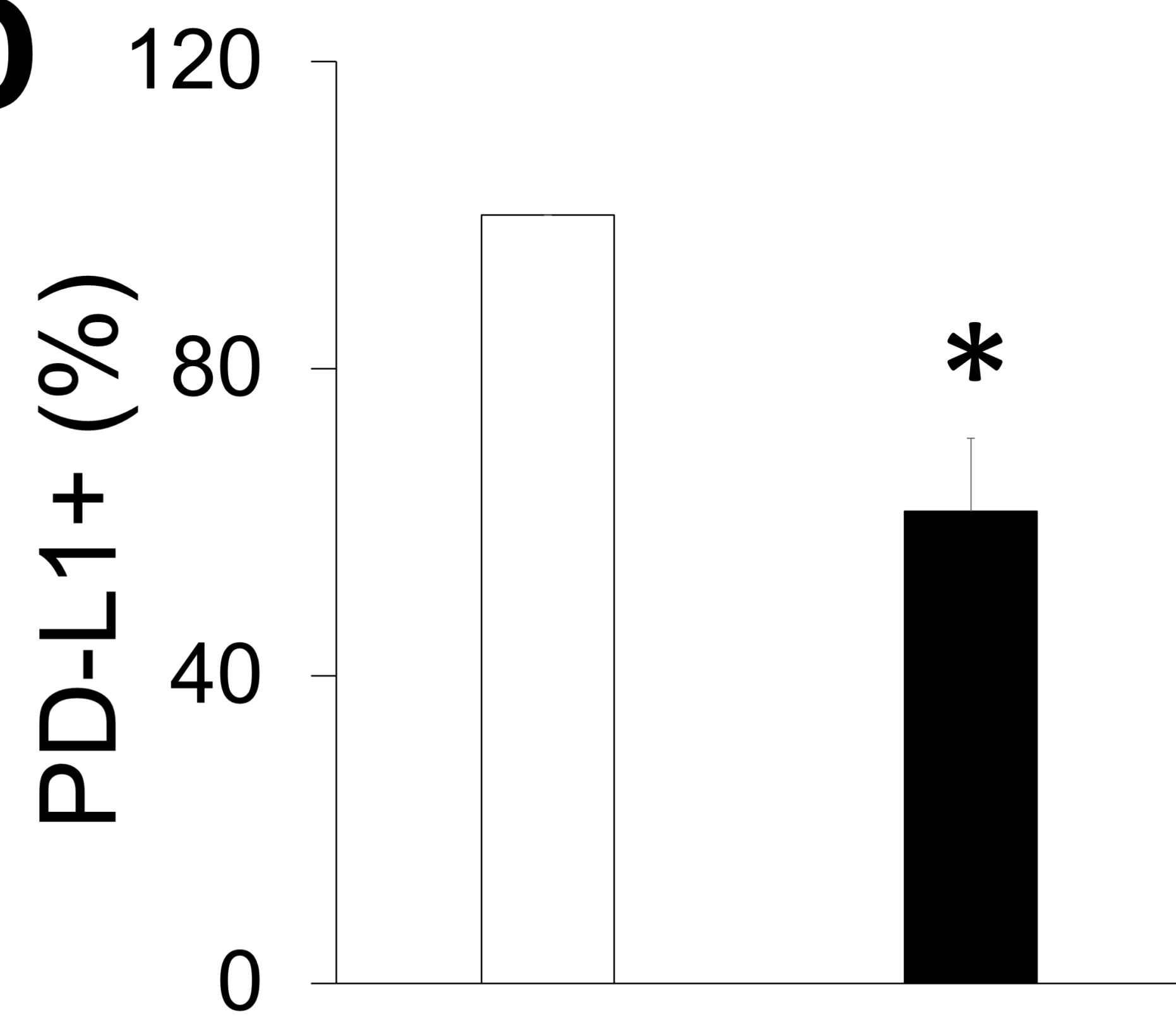**E**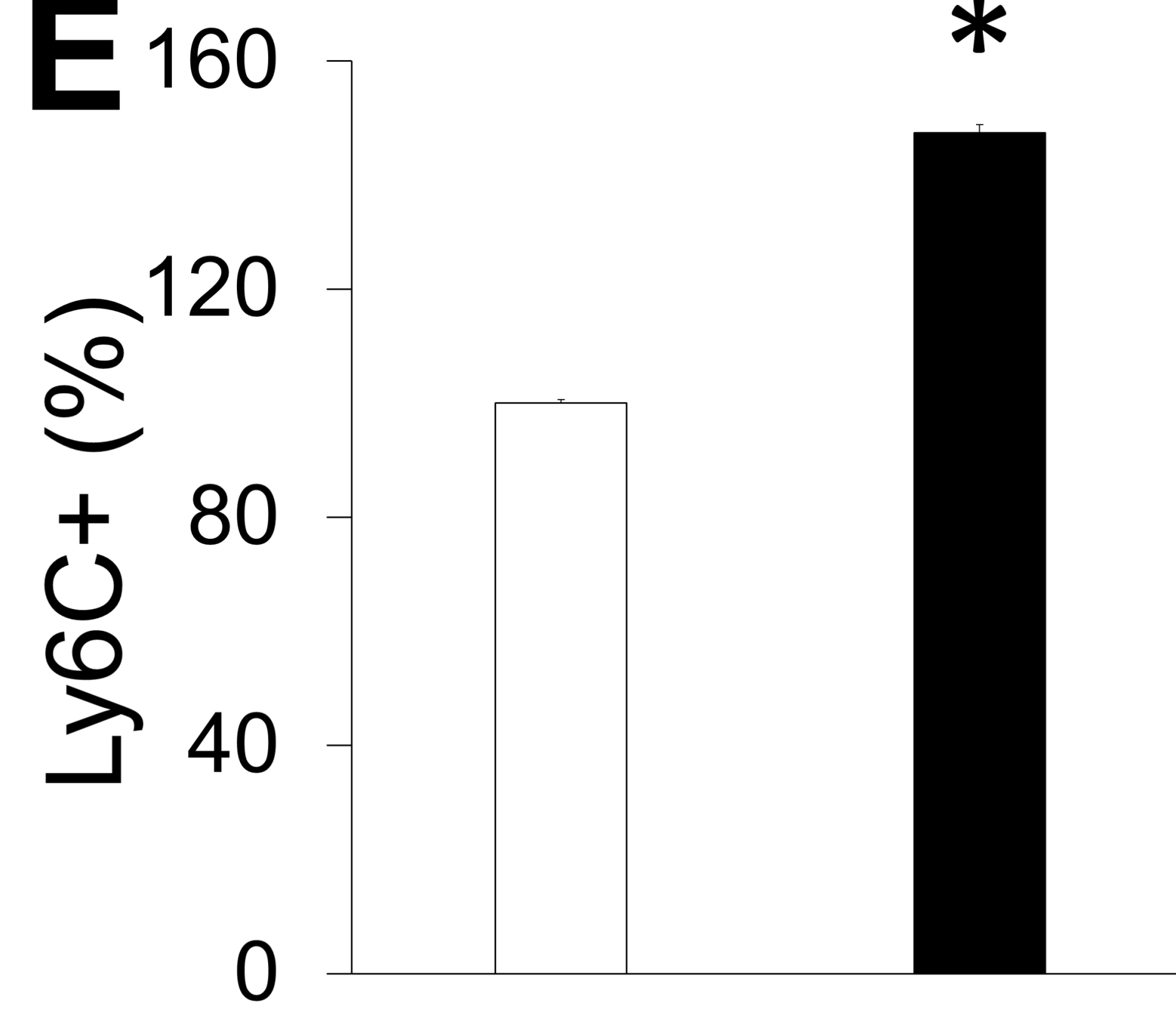**F**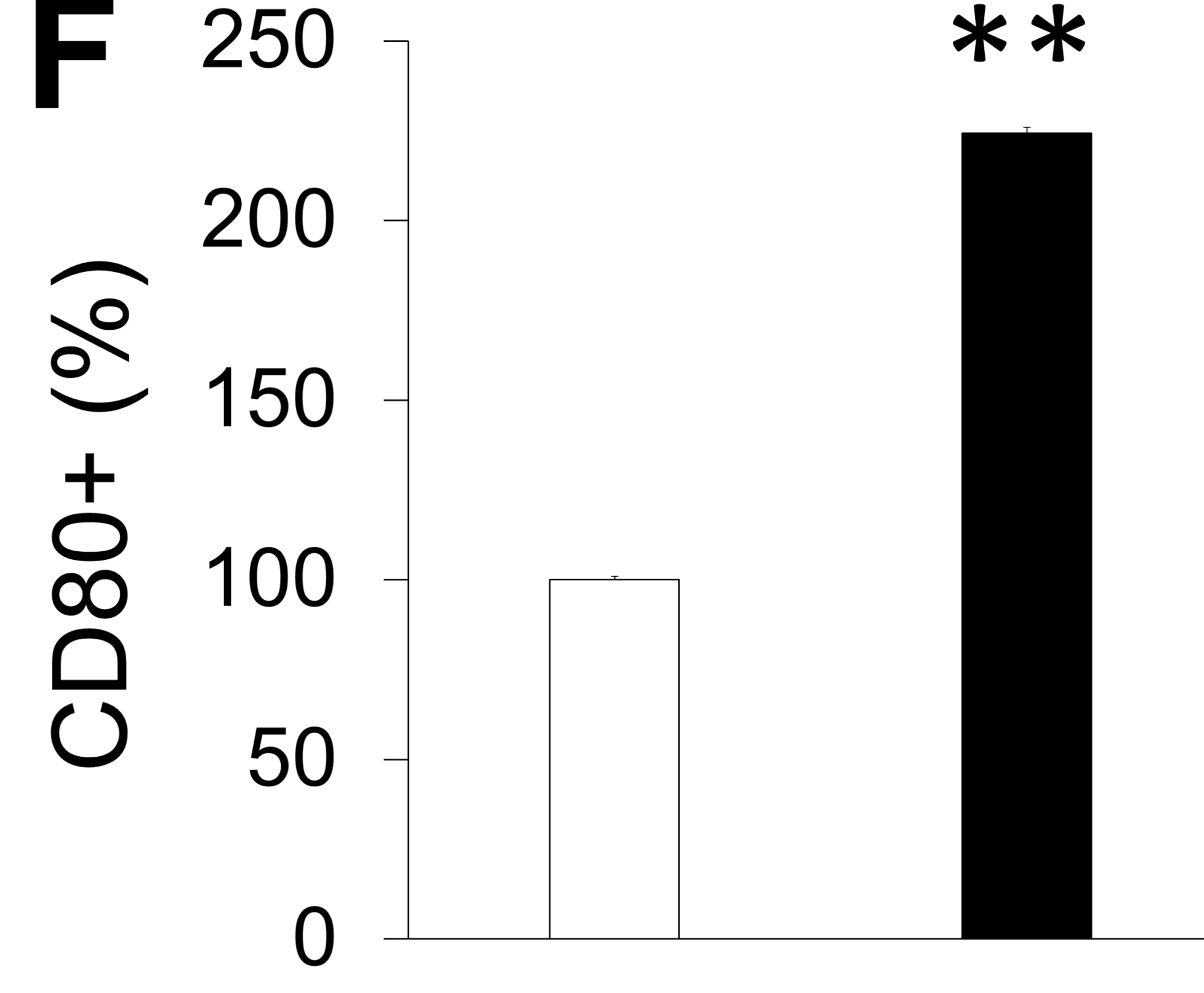**G**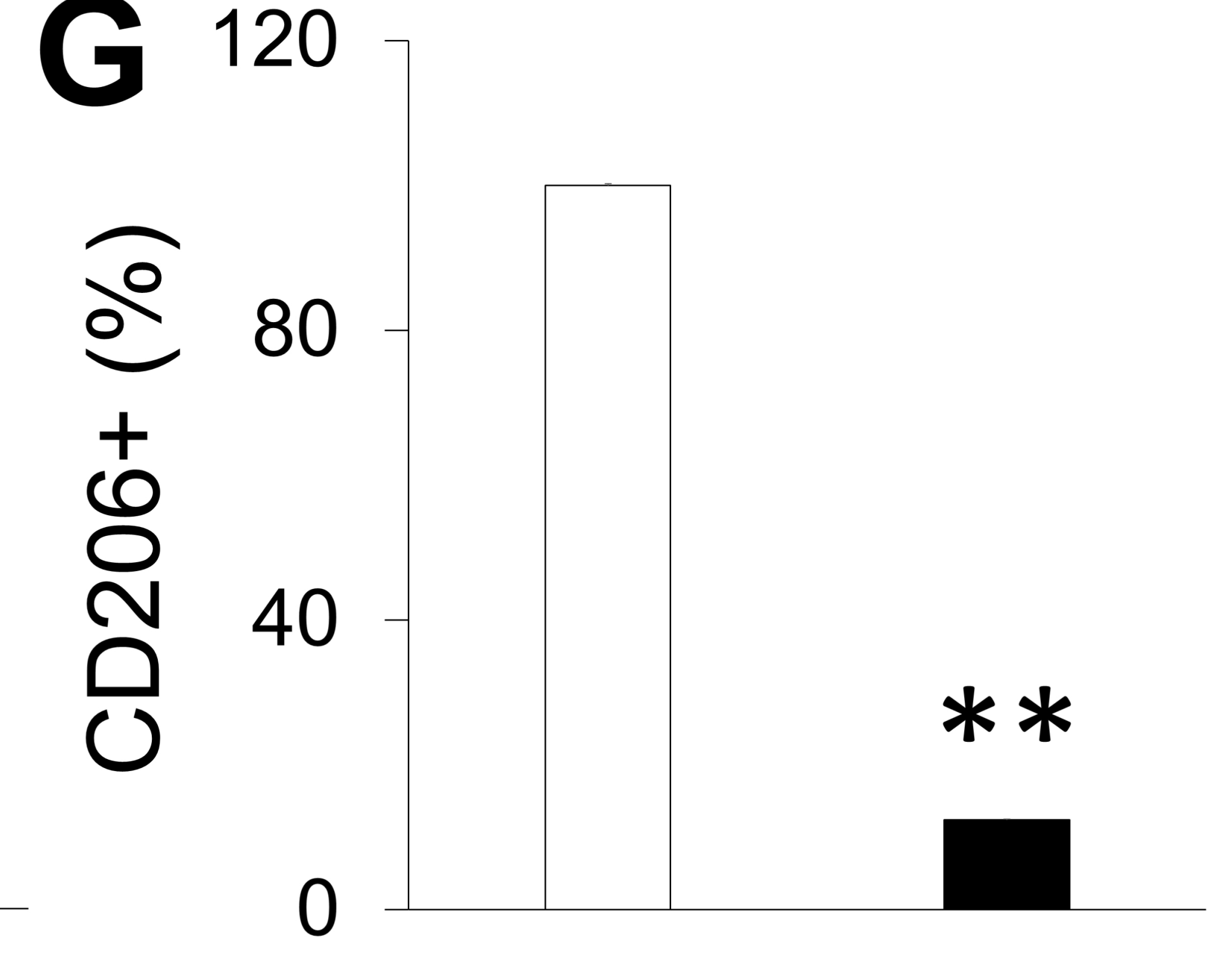

□ Ctl    ■ g82/165+HDR

Supplement: Supplementary file 12 — Supplementary Figure 8. [file 41598_2022_6430_MOESM12_ESM.pdf]

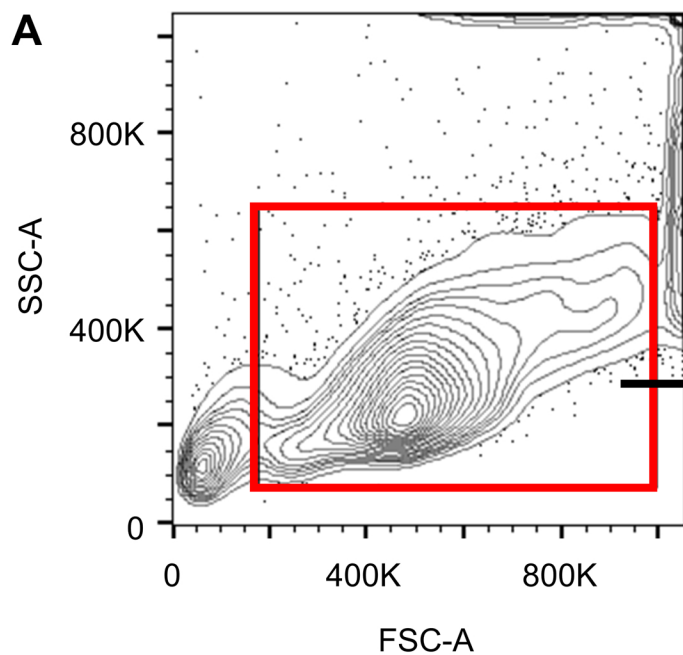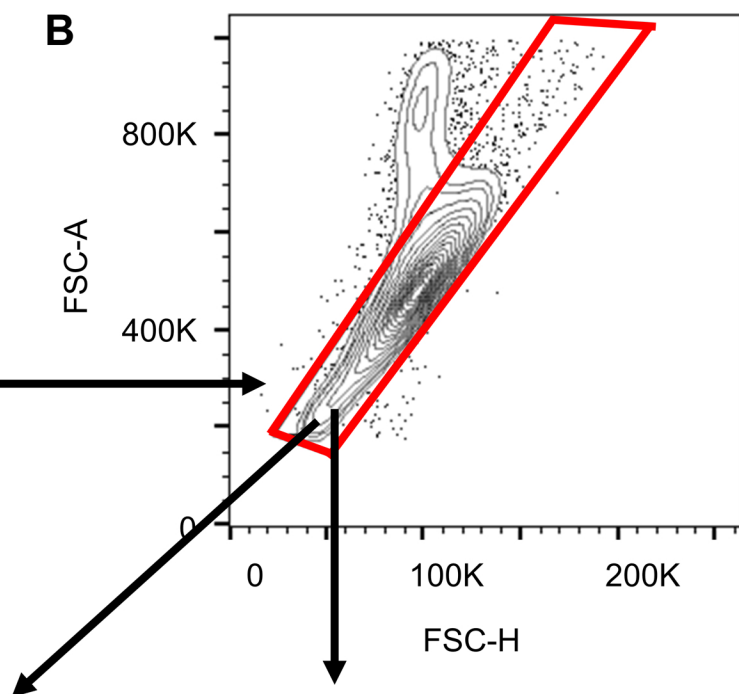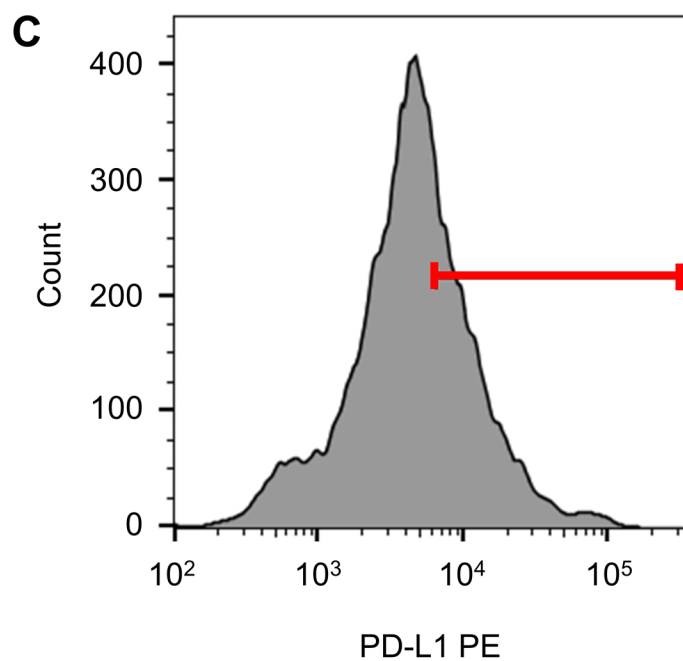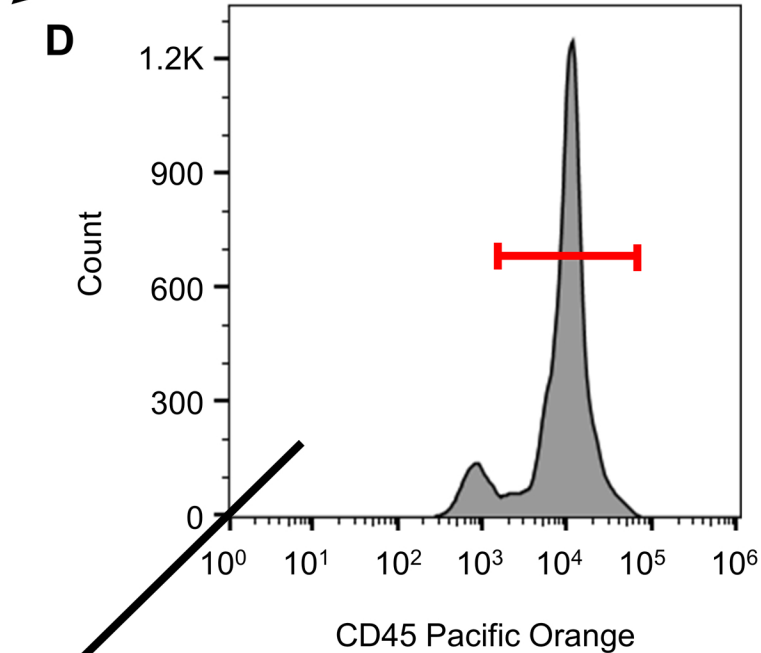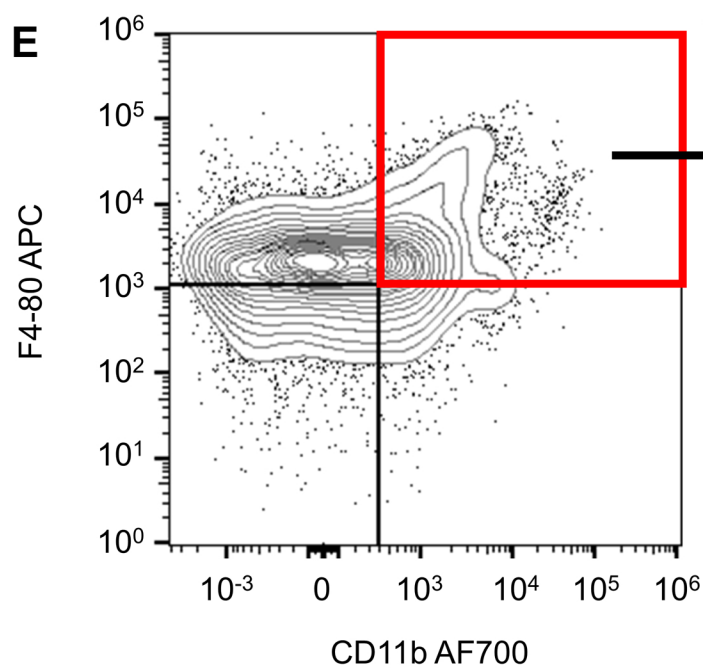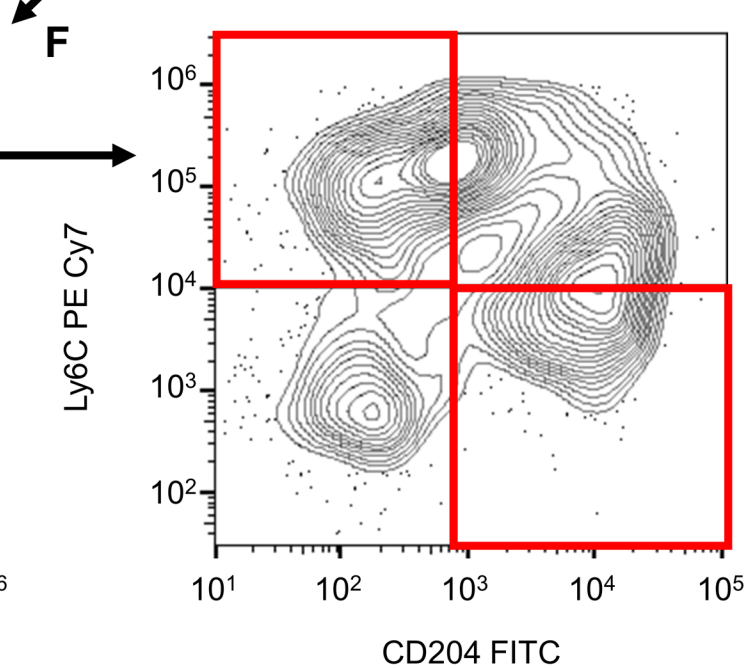

Supplement: Supplementary file 13 — Supplementary Figure 9. [file 41598_2022_6430_MOESM13_ESM.pdf]
