# Supplementary material for: Dual-sgRNA CRISPR/Cas9 knockout of PD-L1 in human U87 glioblastoma tumor cells inhibits proliferation, invasion, and tumor-associated macrophage polarization
Source: Sci Rep. 2022 Feb 14;12:2417. doi: 10.1038/s41598-022-06430-1 (PMC8844083; doi:10.1038/s41598-022-06430-1)
Supplement: Supplementary file 14 — Supplementary Legends. [file 41598_2022_6430_MOESM14_ESM.docx]

**Supplementary data**

**Supplemental Table 1. Off-target analysis for human g82.** Off-target analysis was done using IDT’s CRISPR/Cas9 gRNA design tool. The score indicates the potential for editing at the locus provided, with lower scores indicating a higher potential for editing. #MM indicates the number of DNA bp mismatches from the original gRNA sequence. Human g82 does not target any other cording region.

**Supplemental Table 2: Off-target analysis for human g165.** Off-target analysis was done using IDT’s CRISPR/Cas9 gRNA design tool. The score indicates the potential for editing at the locus provided, with lower scores indicating a higher potential for editing. #MM indicates the number of DNA bp mismatches from the original gRNA sequence. Human g165 does not target any other cording region.

**Supplemental Table 3. Off-target analysis for mouse g70.** Off-target analysis was done using IDT’s CRISPR/Cas9 gRNA design tool. The score indicates the potential for editing at the locus provided, with lower scores indicating a higher potential for editing. #MM indicates the number of DNA bp mismatches from the original gRNA sequence. Mouse g70 does not target any other cording region.

**Supplemental Table 4: Off-target analysis for mouse g166.** Off-target analysis was done using IDT’s CRISPR/Cas9 gRNA design tool. The score indicates the potential for editing at the locus provided, with lower scores indicating a higher potential for editing. #MM indicates the number of DNA bp mismatches from the original gRNA sequence. Mouse g166 does not target any other cording region.

**Supplemental Figure 1. Cloning strategy of *PD-L1* dual-sgRNAs into the CRISPR/Cas9 plasmid.** **(A)** Annealed oligonucleotides for g82 and g165 were cloned into pX330A1x2 and pX330S-2, respectively, by BbsI digestion. These vectors contain a U6 promoter (pink arrow), the gRNA sequence (pink) directly downstream of the cloned oligonucleotides, Cas9 (teal) under a CBh promoter (teal arrow), and either the amp resistance gene (blue; pX330A1x2) or spectinomycin resistance gene (orange; pX330S-2) for selective screening. **(B)** Plasmid DNA for each clone was mixed with BsaI and T4 ligase to generate the Multiplex CRISPR/Cas9 Plasmid via Golden Gate Assembly. Plasmid DNA for Cas9-g82, Cas9-g165, Cas9-g82/165 and Cas9-g82/165+HDR were transfected into U87 cells for studying PD-L1 knockout efficiency. Image inspired by Sakuma *et al* 2014.

**Supplemental Figure 2. Optimization of PD-L1 knockout by Cas9-g82/165+HDR.** Full western blot images showing blots stained for (**A**) FLAG, and (**B**) Actin as seen in figure 2B. L = Ladder in kilo Dalton (kDa); Lane 1 = Lipofectamine only; Lane 2 = 1ug/mL Cas9-g82/165 + 12.5uM HDR; Lane 3 = 1ug/mL Cas9-g82/165 + 25uM HDR; Lane 4 = 1.5ug/mL Cas9-g82/165 + 12.5uM HDR; Lane 5 = 1.5ug/mL Cas9-g82/165 + 25uM HDR; Lane 6 = 2ug/mL Cas9-g82/165 + 12.5uM HDR; Lane 7 = 2ug/mL Cas9-g82/165 + 25uM HDR; Lane 8 = 2.5ug/mL Cas9-g82/165 + 12.5uM HDR; Lane 9 = 2.5ug/mL Cas9-g82/165 + 25uM HDR.

**Supplemental Figure 3. PD-L1 knockout in U87 cells by CRISPR/Cas9 plasmids.** Full western blot images as seen in Figure 3A for (**A**) PD-L1, (**B**) Actin, and (**C**) FLAG. L = Ladder in kilo Dalton (kDa); Lane 1 = Lipofectamine only; Lane 2 = Cas9-g82; Lane 3 = Cas9-g165; Lane 4 = Cas9-g82/165; Lane 5 = Cas9-g82/165 + HDR.

**Supplemental Figure 4. PD-L1 knockout in MDA MB 231 breast cancer cells and in nuclear and cytoplasmic/membrane fractions from U87 cells.** Full western blot images from breast cancer cells as seen in Figure 3D for (**A**) PD-L1, (**B**) FLAG and (**C**) actin. L = Ladder in kilo Dalton (kDa); Lane 1 = Control MDA MB 231 Cells (Lipofectamine only); Lane 2 = Cas9-g82/165+HDR treated MDA MB 231 Cells. Full western blot images are also shown for nuclear/cytoplasmic protein extractions from U87 GBM cells as seen in Figure 3I for (**C**) actin (lanes 3 – 6) (**D**) PD-L1, and (**E**) histone H3. Lane 3 = control, membrane/cytoplasm fraction; Lane 4 = control, nuclear fraction; Lane 5 = Cas9-g82/165+HDR, membrane/cytoplasm fraction; Lane 6 = Cas9-g82/165+HDR, nuclear fraction

**Supplemental Figure 5. U87 cell growth after PD-L1 knockout.** The growth of U87 cells transfected with or without Cas9-g82/165+HDR were monitored over 3 days. Images revealed that cells treated with Cas9-g82/165+HDR grew slower than control cells.

**Supplemental Figure 6. Gating strategy for flow cytometry for U87 cells co-cultured with human SC macrophages.** (**A**) Debris was first removed by examining the forward (FSC-A) and side scatter (SSC-A) plots. (**B**) Next, single cells were identified by examining the forward scatter area (FSC-A) and forward scatter height (FSC-H) plots. Cells were then gated to identify PD-L1 knockout in U87 cells ((**C**) CD45- (**D**) CD11b-PD-L1+). (**E**) CD45+CD11b+ cells were gated for (**F**) CD80+Arginase1- M1 cells, and Arginase I+CD80- M2 cells.

**Supplemental Figure 7. Gating strategy for flow cytometry for U87 cells co-cultured with mouse bone marrow derived macrophages.** (**A**) Debris was first removed by examining the forward (FSC-A) and side scatter (SSC-A) plots. (**B**) Next, single cells were identified by examining the forward scatter area (FSC-A) and forward scatter height (FSC-H) plots. Cells were then gated to identify PD-L1 knockout in U87 cells ((**C**) CD45- (**D**) CD11b-PD-L1+). (**E**) CD45+CD11b+ cells were gated to identify (**F**) CD80+/CD206- and (**G**) Ly6C+Ly6G- M1 cells, and (**F**) CD206+/CD80- M2 cells.

**Supplemental Figure 8: PD-L1 knockout in U87 cells polarizes TAMs to M1.** U87 cells were treated with Cas9-g82/165+HDR for 24 hours, then bone marrow derived macrophages were added allowing TAM differentiation for 48 hours. The co-cultures were collected and analyzed by flow cytometry. Contour plots of **(A)** CD11b and PD-L1, **(B)** Ly6C and Ly6G**,** and **(C)** CD206 and CD80 subpopulations are shown for Cas9-g82/165+HDR treated cultures and the control. **(D)** Co-cultivation of TAMs and U87 cells treated with Cas9-g82/165+HDR significantly decreased CD45-CD11b-PD-L1+ subpopulations compared to the control, suggesting Cas9-g82/165+HDR was able to knockout PD-L1 in U87 cells. PD-L1 knockout in U87 cells repolarized M2 TAMs into **(E)** Ly6C+ and **(F)** CD80+ M1 TAMs. In contrast, PD-L1 deletion in U87 cells leads to **(G)** CD206+ M2 depletion. All experiments were performed in triplicate and results were normalized for direct comparisons. Data represents the mean ± SEM. Statistical significance was evaluated with a t-test. * *p<0.05*, ** *p<0.01*.

**Supplemental Figure 9. Gating strategy for flow cytometry for *in vivo* studies.** (**A**) Debris was first removed by examining the forward (FSC-A) and side scatter (SSC-A) plots. (**B**) Next, single cells were identified by examining the forward scatter area (FSC-A) and forward scatter height (FSC-H) plots. (**C**) Cells were then gated to identify total PD-L1 knockout in splenocytes (PD-L1+). (**D**) CD45+CD11b+ cells were further gated to identify (**E**) CD11b+F4/80+ macrophages, which were then categorized into (**F**) Ly6C+CD204- M1 cells and CD206+Ly6C- M2 cells.
